# COSMOPharm: Drug–Polymer Compatibility of Pharmaceutical Amorphous Solid Dispersions from COSMO-SAC

**DOI:** 10.1021/acs.molpharmaceut.4c00342

**Published:** 2024-07-30

**Authors:** Ivan Antolović, Jadran Vrabec, Martin Klajmon

**Affiliations:** † Thermodynamics, Technische Universität Berlin, Ernst-Reuter-Platz 1, 10587 Berlin, Germany; ‡ Department of Physical Chemistry, University of Chemistry and Technology, Prague, Technická 5, 166 28 Prague 6, Czechia

**Keywords:** amorphous solid dispersions (ASD), drug−polymer thermodynamic compatibility, solubility, miscibility, prediction, COSMO-SAC, quantum mechanics

## Abstract

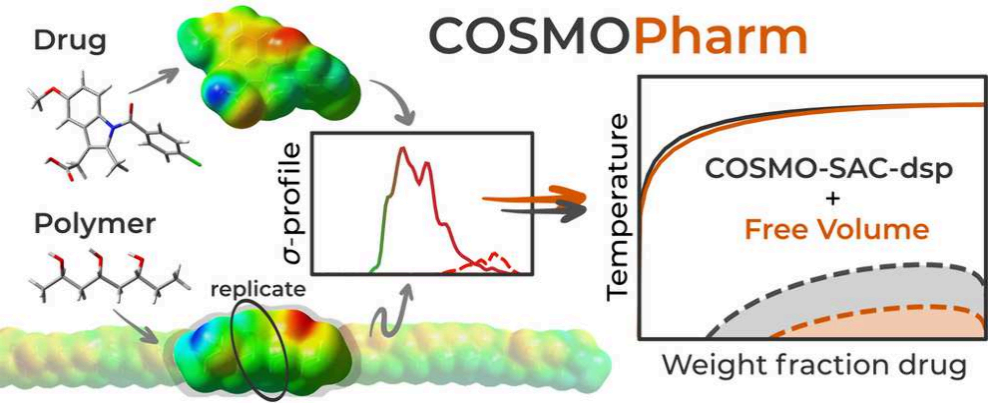

The quantum mechanics-aided COSMO-SAC activity coefficient model is applied and systematically examined for predicting the thermodynamic compatibility of drugs and polymers. The drug–polymer compatibility is a key aspect in the rational selection of optimal polymeric carriers for pharmaceutical amorphous solid dispersions (ASD) that enhance drug bioavailability. The drug–polymer compatibility is evaluated in terms of both solubility and miscibility, calculated using standard thermodynamic equilibrium relations based on the activity coefficients predicted by COSMO-SAC. As inherent to COSMO-SAC, our approach relies only on quantum-mechanically derived σ-profiles of the considered molecular species and involves no parameter fitting to experimental data. All σ-profiles used were determined in this work, with those of the polymers being derived from their shorter oligomers by replicating the properties of their central monomer unit(s). Quantitatively, COSMO-SAC achieved an overall average absolute deviation of 13% in weight fraction drug solubility predictions compared to experimental data. Qualitatively, COSMO-SAC correctly categorized different polymer types in terms of their compatibility with drugs and provided meaningful estimations of the amorphous–amorphous phase separation. Furthermore, we analyzed the sensitivity of the COSMO-SAC results for ASD to different model configurations and σ-profiles of polymers. In general, while the free volume and dispersion terms exerted a limited effect on predictions, the structures of oligomers used to produce σ-profiles of polymers appeared to be more important, especially in the case of strongly interacting polymers. Explanations for these observations are provided. COSMO-SAC proved to be an efficient method for compatibility prediction and polymer screening in ASD, particularly in terms of its performance–cost ratio, as it relies only on first-principles calculations for the considered molecular species. The open-source nature of both COSMO-SAC and the Python-based tool COSMOPharm, developed in this work for predicting the API–polymer thermodynamic compatibility, invites interested readers to explore and utilize this method for further research or assistance in the design of pharmaceutical formulations.

## 1 Introduction

Most newly produced drugs are poorly soluble in water due to their pronounced hydrophobic character. This often significantly limits their bioavailability, particularly at the dissolution stage after oral administration. To overcome this issue, various methodologies have been developed.^1^ One of the most efficient techniques is the formulation of an amorphous solid dispersion (ASD), where an active pharmaceutical ingredient (API) is molecularly dispersed in a suitable polymeric carrier.^2^ The conversion of a crystalline API into its amorphous form leads to a higher solubility (because the latter form has a higher Gibbs energy), while its dispersion in a polymer entails better stability preventing recrystallization (because chain-like polymer molecules restrict the translation of dispersed API molecules).^3−5^ However, such a stabilization is only temporary, if the API–polymer solution is supersaturated with respect to the API. In such cases, an amorphous–amorphous phase separation (AAPS) may occur over time, followed by the nucleation and recrystallization of the API. It is thus important to carefully select an appropriate polymer that has a favorable thermodynamic compatibility with a given API.^6^ The term *compatibility* covers both miscibility and solubility of an API with respect to the polymeric carrier, which are macroscopic thermodynamic phase behavior properties described by means of liquid–liquid equilibria (LLE; i.e., AAPS) and solid–liquid equilibria (SLE), respectively. Knowledge of the relation between temperature and composition under SLE and LLE of an API–polymer system allows for the construction of an equilibrium phase diagram from which the thermodynamic compatibility between an API and a polymer under various conditions can be deduced. In this way, optimal polymeric carrier(s) for a given API are rationally designed.

**Figure 1:**
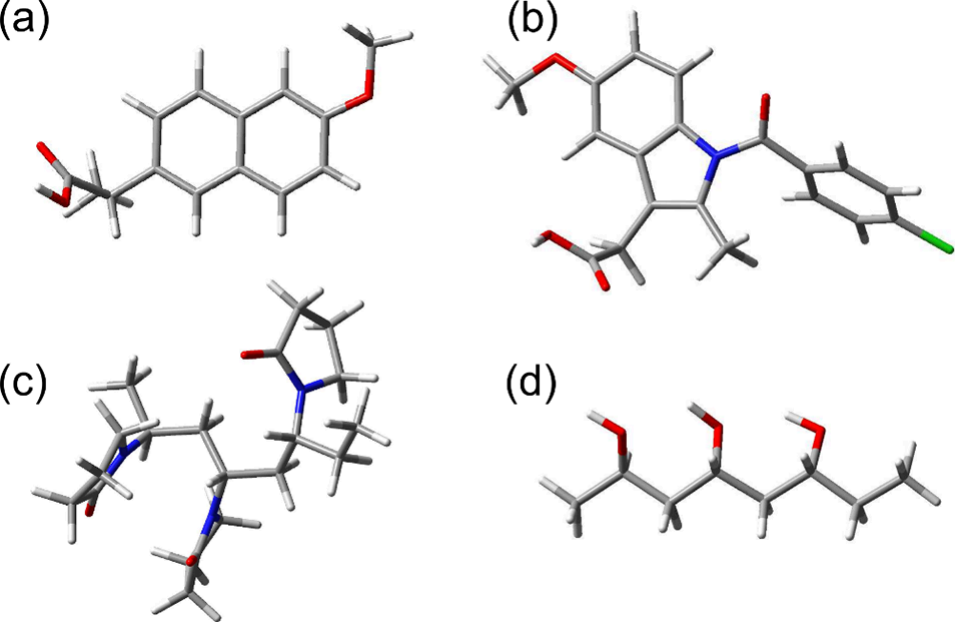
Molecular structures of representative API: (a) naproxen (abbreviated NPX) and (b) indomethacin (IMC); along with trimers of representative polymers: (c) poly(vinylpyrrolidone) (PVP) and (d) poly(vinyl alcohol) (PVA).

The design of drug delivery systems including polymer-based ASD is still governed by trial-and-error approaches, which particularly include a laborious experimental program. However, to reduce the financial expenditure and speed-up ASD design processes, predictive computational approaches can be employed.^7^ Various theoretical frameworks exist for drug–polymer systems, differing in their theoretical background, level of insight, performance, and parametrization effort in terms of required input information (typically experimental data). Therefore, it is useful to provide an overview of the current state in the field of computational approaches to ASD that pharmaceutical formulators currently have at hand to predict API–polymer compatibility. Before we do so, it is important to note that API–polymer systems are generally very challenging to model because of the structural and interactional complexity of the relevant molecular components. They possess diverse functional groups that can participate in different molecular interactions. Structures of some API and polymers are shown in Figure 1. Most importantly, ASD are typically hydrogen-bonded systems in which both inter- and intramolecular hydrogen bonds (HB) can form.^8^ Accordingly, the degree of API–polymer compatibility is often attributed to the strength of the respective HB interactions.^9,10^ In addition to HB, π–π stacking and van der Waals interactions are also important factors in the context of ASD. Considering the intricate complexities of both API and polymer molecules and recognizing that their mutual thermodynamic compatibility is significantly influenced by their interactions in the liquid phase (captured by the activity coefficients), it becomes evident that the ability of a model to account for specific structural and interactional characteristics is a crucial determinant for its power in predicting the API–polymer compatibility.

Starting with the most fundamental techniques, quantum mechanical (QM) calculations are generally limited to systems consisting of a relatively small number of atoms, because of their computational expense. Although they are invaluable, for example, in mapping interactions of API with biomolecules,^11,12^ coformers,^13^ or polymers^9,10^ for computer-aided drug and formulation design, they currently cannot be efficiently used in a stand-alone manner to obtain macroscopic phase behavior of bulk API–polymer systems. Among molecular-mechanical (MM) approaches, molecular dynamics (MD) simulations are valuable for investigating the transport, structural, and thermodynamic properties of bulk API,^14−17^ polymers,^18,19^ and API–polymer mixtures.^20−23^ However, MD simulations of polymer systems can also be computationally expensive, and their outcomes appear to be sensitive to the simulation approach, force field (FF) selection, and post-processing of the sampled trajectories.^19^ As the latest trend, machine learning (ML)-enhanced techniques for simulations of material properties, such as active Δ-ML, are being developed.^24−27^ These methods use highly accurate FF models trained on-the-fly, combining the accuracy of QM methods with the computational efficiency of classical FF. However, as far as we know, these novel methods have not yet penetrated the field of ASD.

Among semiempirical, more “engineering-like” approaches, an important class of models rests on the excess Gibbs energy (*G*^E^). The first successful theory for modeling polymer systems was the Flory–Huggins model,^28,29^ which was later also applied to ASD.^21,30−32^ In addition to the volume of the involved molecular species required for the combinatorial (i.e., entropic) contribution to *G*^E^, the central point of its performance for non-athermal mixtures appears to be the parameter χ_*ij*_ that primarily quantifies the magnitude of the interaction energy between unlike components *i* and *j* (i.e., enthalpic contribution). It is usually fitted to experimental mixture data, but can also be estimated computationally. However, the latter option appears to show serious failures, at least for API–polymer systems.^21^ The most fundamental problem of this model stems from the absence of an explicit treatment of specific intermolecular interactions such as HB,^8^ which are particularly important in the context of ASD. Nevertheless, the combinatorial Flory–Huggins term serves as a basis of many more elaborate *G*^E^ models applied to pharmaceutical systems, including the UNIFAC^33^ and COSMO^34^ frameworks. Finally, the Hansen solubility parameter approach, being able to estimate χ_*ij*_, *G*^E^, or the compatibility itself directly, remains popular for API–excipient formulations despite its limitations.^21,35^

Another important group of semiempirical tools is constituted by equations of state (EOS). One of the most popular EOS for modeling the phase behavior of polymer, pharmaceutical, and ASD systems is PC-SAFT.^32,36−41^ Unlike the Flory–Huggins and UNIFAC models, EOS like PC-SAFT are capable of explicitly describing HB. They typically require experimental data for parametrization of the involved molecular species (i.e., pure component parameters) and, for better accuracy, also for mixtures (binary interaction parameters, *k*_*ij*_). It was recently shown that predictions of the API solubility in polymers obtained from PC-SAFT without any mixture-specific parameters (i.e., with all *k*_*ij*_ set to zero) achieve an overall average absolute relative deviation of approximately 50%^41^ in terms of weight fraction solubility (statistical parameters are explained in Section 2.4). Regardless of the quantitative performance, the qualitative ranking of polymers with respect to predicted compatibility with API showed good results. Because of their semiempirical nature, a characteristic aspect of both EOS and traditional *G*^E^ models is that they heavily rely on experimental data to regress their parameters, and that their results for systems and conditions that were not included in their parametrization may have limited reliability. However, an important advantage of EOS is that they provide in addition to the activity coefficients also other thermodynamic properties as a function of temperature, density, and composition (although this feature is rarely exploited in the context of pharmaceutical systems^39^).

Predictive group-contribution (GC) approaches within both *G*^E^ models and EOS provide great flexibility in describing various types of systems. However, this critically depends on the availability of the parameters for the involved functional groups, which are often missing even for the leading frameworks, such as the various versions of the modified UNIFAC model^33,42^ and the SAFT-γ-Mie EOS.^43^ The complex multifunctional nature of molecules that are of pharmaceutical interest (API, polymers, etc.) contributes to this issue. For example, as recently emerged from a survey performed by Kontogeorgis et al.,^44^ the number of API currently studied by pharmaceutical companies whose molecules can be fully incremented by GC models and covered by their parameter matrices is practically limited to zero. This explains why GC models are widely applied in problems relevant to, e.g., the oil and gas industry, but have never become established in pharmaceutical or formulation communities.^35^ Another drawback of GC models is that they typically assume that functional groups have invariant properties across different chemical environments, although this assumption is not always true.^45^ For example, the carboxyl group acts differently in acetic acid than in naproxen. However, new ML-based approaches might solve these problems and fill the gaps in group interaction parameter matrices in the near future.^46^ With ML approaches, efforts are underway to develop models for the design of ASD.^10,47,48^ These models, once trained on a substantial dataset comprising experimental or experiment-based information on API–polymer systems and equipped with ten or more molecular descriptors for each API–polymer pair under investigation, show promising results.

Most approaches to obtain the macroscopic phase behavior of ASD, namely, *G*^E^, EOS, GC, and ML models, rely on more or less challenging parametrization procedures to adjust the model parameters to experimental data. Conversely, COSMO-based models, such as the original COSMO-RS (conductor-like screening model for real solvents)^34^ and COSMO-SAC (COSMO segment activity coefficient),^49,50^ are strictly predictive. They combine QM single-molecule calculations of molecular electrostatic properties with a statistical thermodynamic approach to estimate the macroscopic thermodynamic properties of solutions including the activity coefficients and, consequently, phase equilibria. The QM calculations are performed assuming a continuum solvation model to simulate the presence of a solvent environment and its influence on the molecular properties. In principle, COSMO-based models only require the molecular structure of the studied compounds as an input and do not rely on any substance- or mixture-specific experimental data. As such, they represent an efficient framework for a priori predictions of the activity coefficients and macroscopic phase equilibria of different types of solutions, including API and polymers.

The theoretically sound nature of COSMO-based models together with the practical absence of parametrization effort make them popular in various fields, including pharmaceutical applications related to different stages of drug development, solvent screening, excipient ranking, and formulation design.^51−54^ For example, they can be applied to the classical problem of the solubility of API in conventional low-molar-mass (low-*M*) solvents^17,52,55−57^ as well as in those with higher *M* (but not higher than ∼1000 g mol^–1^).^51,54^ They have also been applied to polymer systems, specifically, to mixtures of a polymer and a low-*M* solvent, gas, or ionic liquid.^58−64^ It should be noted that the application of COSMO-based models to polymer systems requires special treatments that introduce certain challenges and, at the same time, degrees of freedom in the modeling approach, as described in Section 2.2. Almost all of the cited applications aimed at evaluating and illustrating the predictive power of the COSMO-type models and typically reported encouraging results. It is therefore not surprising that they are nowadays considered to be an established tool for high-throughput screening purposes in pharmaceutical practice.^35,53^ However, it is somewhat surprising that, to the best of our knowledge, there is no publicly available application of COSMO-based models to mixtures of API with polymers. Therefore, it remains to be explored as to what is the performance of COSMO-based models for ASD. To introduce the first application of them to ASD in this work, we opt for the COSMO-SAC model,^49,50^ due to its fully documented background and open-source nature, which are attributes that the ASD community can leverage.

This work aims to investigate the capabilities and reliability of activity coefficients predicted by COSMO-SAC for screening the API–polymer compatibility. The goal is to illustrate what can be expected from the open-source COSMO-SAC implementation^50^ when used in screening for suitable polymer candidates for a given API. It is important to note that this approach (and the COSMO-SAC approach itself, in general) includes no parameter training to API, polymer, or API–polymer experimental data. With a carefully explained methodology together with public availability of most of the employed computational tools, this document may also serve as a guide for formulators that do not wish to rely on commercial software tools or models requiring experimental data for their parametrization and performance.

As an exemplary sample, a set of seven API, nine polymers (both homo- and copolymers), and 35 different API–polymer systems thereof is considered, for which consistent and reliable experimental solubility data were measured by Mathers and co-workers in Prague in recent years.^40,65−67^ These data were used to evaluate the performance of COSMO-SAC. An overview with chemical identifiers of the API and polymers considered in this work is given in Tables S1 and S2, respectively, in the Supporting Information (SI).

To complement the theoretical advancements and practical applications discussed, we introduce COSMOPharm, a tool developed to harness the predictive capabilities of COSMO-SAC for evaluating the API–polymer thermodynamic compatibility. Designed with the user in mind, COSMOPharm aims to make the insights gained from COSMO-SAC accessible to researchers and formulators, enabling efficient screening of polymers for drug formulation.

The next section describes the methodological and computational details, including the procedure to obtain the σ-profiles for API and polymers. The prediction results obtained from COSMO-SAC are then presented and comprehensively discussed in Section 3. The findings are summarized and contextualized in the last section.

## 2 Computational Methods

This section begins with a description of the thermodynamic equilibrium relations used to calculate the solubility and miscibility of ASD. Subsequently, the COSMO-SAC model is presented, along with the methodology employed to handle API and polymers with that model. Finally, the sources of reference experimental data are provided.

### 2.1 Thermodynamic Solubility and Miscibility Calculations

The solubility of a solid crystalline API, represented by the mole fraction of API (*x*_API_) in a saturated liquid (amorphous) phase at a temperature *T*, is expressed through the SLE equation^68^
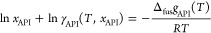
1The term ln γ_API_ contains the activity coefficient of the API in solution, which considers the non-ideality of the liquid mixture and has to be computed with a thermodynamic model, e.g., COSMO-SAC. The symbol *R* denotes the universal gas constant. Furthermore, Δ_fus_*g*_API_ signifies the molar Gibbs energy difference between the standard state, that is, the pure supercooled liquid API, and the pure crystalline form of the API. The calculation of Δ_fus_*g*_API_ is based on the thermodynamic properties of the pure API, employing the rigorous thermodynamic relation
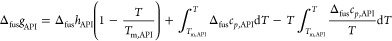
2where *T*_m,API_ denotes the melting point of the API, Δ_fus_*h*_API_ refers to the molar enthalpy of fusion of the API at its melting point, and Δ_fus_*c*_*p*,API_ represents the difference in isobaric heat capacity between the liquid and crystalline phases of the pure API.

Note that ln γ_API_ is the sole quantity in eq 1 determined with COSMO-SAC and is also the sole quantity that describes the influence of the specific polymer. Moreover, eq 1 requires data for *T*_m_, Δ_fus_*h*, and Δ_fus_*c*_*p*_ of the pure API. In this work, established experimental data were used for this purpose, as listed in Table 1. If experimental data are unavailable, various alternative methods can be employed to estimate these properties with varying accuracy.^69−72^

**Table 1:** Thermodynamic Properties of the Considered API

API	Form^a^	*T*_m_/K	Δ_fus_*h*/(kJ mol^–1^)	Δ_fus_*c*_*p*_/(J K^–1^ mol^–1^)	Source^b^
GSF	I	491.85	37.90	93.84	refs (67, 73)
IBP	I	348.55	26.40	176.16440 – 0.3449480 · (*T*/K)	ref (74)
IMC	γ	433.35	38.10	238.18385 – 0.2785901 · (*T*/K)	ref (74)
NIF	α	445.75	39.30	121.22	refs (67, 75)
NPX	I	429.25	32.40	99.30	ref (74)
PCM	I	442.55	28.00	99.80	refs (76, 77)
SIM	I	412.45	27.75	278.77100 – 0.331300 · (*T*/K)	refs (67, 78)

a The specific API polymorph form considered in this work.

b When citing two sources, the second one pertains to Δ_fus_*c*_*p*_.

A solubility curve of an API in a polymer under SLE was generated on the basis of a series of *x*_API_ values computed with eq 1 (with ln γ_API_ determined with COSMO-SAC) as a function of temperature. The resulting mole fraction values *x*_API_ were then converted to weight fractions *w*_API_, which were utilized to depict phase diagrams and evaluate the accuracy of COSMO-SAC predictions.

In the context of systems that show AAPS, two methodologies to calculate the LLE are prevalent: the direct numerical solution approach and the application of the alternating tangents concept proposed by von Solms et al.^79^ These approaches are fundamentally distinct in their operational mechanisms. The direct numerical solution concurrently resolves the equilibrium conditions for both phases, whereas the alternating tangents method sequentially addresses the equilibrium of each phase. The present investigation initially employed both methods for comparative analyses, and predominantly utilized the direct numerical solution approach. It is imperative to note that irrespective of the chosen method, the calculated binodal points must rigorously adhere to the standard LLE equilibrium condition. This condition mandates the equality of the chemical potential for each component across the liquid phases, as delineated by the isoactivity condition, considering the standard state of pure liquid component at the temperature and pressure of the system for both API and polymer
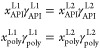
3The superscripts L1 and L2 indicate the two liquid (amorphous) phases involved in the equilibrium. The mole fractions *x*_API_^L1^ and *x*_API_^L2^ represent the binodal points corresponding to these phases. By iteratively solving these equations over a range of temperatures, the mole fractions and activity coefficients are determined. Adjusting the temperature and recalculating the values ensures equilibrium is reached. The resulting binodal points form the binodal curve, outlining the AAPS region.

Unlike the solubility eq 1, ln γ_*i*_ determined with COSMO-SAC is the only input in eq 3. This means that LLE are more sensitive to the modeling approach used to determine ln γ_*i*_ than SLE and vapor–liquid equilibria (VLE), where melting and boiling points of pure components, respectively, act as “anchor” points in the respective phase diagrams.

A general description of the phase diagrams considered for ASD including the SLE and LLE (and also glass-transition temperature, *T*_g_) is provided in Section S2 in the SI, to which the reader is referred to for interpreting the diagrams presented in this paper.

### 2.2 COSMO-SAC

#### 2.2.1 COSMO-SAC Theory

The basic idea of COSMO-type models is a synergistic combination of QM and statistical thermodynamics. Specifically, molecular properties of the species under consideration are calculated quantum-mechanically (namely, the surface screening charge density σ and surface area *A*, both divided into surface segments), and the obtained QM data are used in a statistical approach for the interaction of surface segments that produces the macroscopic properties of a solution, such as *G*^E^ and activity coefficients. For the QM part, density functional theory (DFT) calculations together with a polarizable continuum model for the implicit solvation (usually the COSMO model^80^ or its alternatives) are used.

The “bridge” connecting the QM and statistical thermodynamic parts of COSMO-type models can be seen in the so-called σ-profile of a molecular species, denoted as *p*_*i*_(σ), which represents the most important molecule-specific information in the COSMO framework. It transforms and condenses the initially three-dimensional information on surface screening charge density σ of individual surface segments calculated by QM into a histogram describing the relative distribution of molecular surface areas corresponding to individual σ values. *p*_*i*_(σ) is subsequently used in the statistical surface interaction model. It should be noted that before the transformation to *p*_*i*_(σ), the σ value of each segment undergoes an averaging procedure, considering σ values of the other segments and their mutual distances.^50^

In this work, we used the COSMO-SAC-dsp model, which is a dispersive variant of COSMO-SAC that incorporates electrostatic (misfit), specific HB, and dispersion interactions. This model, proposed by Hsieh et al.,^81^ was implemented in the open-source COSMO-SAC package developed by Bell et al.^50^ Because these two publications provide a thorough description of COSMO-SAC-dsp and the general COSMO-SAC framework, it is focused here only on the essential concepts and equations.

The original COSMO-SAC model by Lin and Sandler^49^ was further improved by accounting for variations in strength of HB interactions using separate σ-profiles. In 2010, Hsieh et al.^82^ introduced the COSMO-SAC-2010 model which included a more detailed approach to HB interactions. Here, the molecular surface is divided into segments that are non-hydrogen-bonding (NHB) and segments that form hydrogen bonds either through bonds of a hydroxyl group (OH) or other hydrogen bonds (OT). This refinement allowed for more accurate predictions, especially for mixtures involving complex HB interactions. Both the COSMO-SAC-2010 and the COSMO-SAC-dsp models incorporate these diverse HB types.

The activity coefficient of the component *i* can be written as the sum of three individual contributions
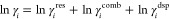
4Therein, the superscript “res” denotes the residual (enthalpic) contribution that describes the electrostatic interactions between the unlike molecules in the mixture based on their σ-profiles. The superscript “comb” then indicates the combinatorial (entropic) contribution that accounts for both size and shape differences between the molecules. In COSMO-based models, the Staverman–Guggenheim (SG) relation is typically employed to determine ln *γ*_*i*_^comb^ (i.e., ln *γ*_*i*_^comb^ = ln *γ*_*i*_^SG^). More details about these two terms can be found, e.g. in ref (50). The last term on right-hand side of eq 4 represents the contribution of the dispersive interactions. Since the description of these ubiquitous attractive interactions was originally missing in the COSMO-based models, Hsieh et al.^81^ introduced an ad hoc dispersion contribution ln *γ*_*i*_^dsp^ for the use with COSMO-SAC by using a single-constant Margules equation. For a binary mixture of components 1 and 2, it reads
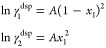
5Therein, the constant *A* is determined from atomic contributions that were predetermined by regressing selected experimental VLE data of conventional mixtures.^81^

It is important to note that COSMO-SAC operates without the need for specific data on the targeted system, and relies only on a set of universal substance-independent parameters. Although these parameters are considered as universally applicable, it should be kept in mind that they were optimized to a small selected set of diverse experimental data^49,81−83^ and considering DFT-based σ-profiles from one particular library.^56,84,85^ However, the experimental set used to determine the universal parameters of COSMO-SAC did not contain any data on SLE or data pertaining to API-like molecules or polymers. Therefore, the prediction of API–polymer SLE is a solid test of the model. The present study can therefore be considered as a continuation of previous research in testing the performance of COSMO-SAC for different types of complex systems.^60,86,87^

#### 2.2.2 Free Volume Term

The combinatorial contribution is the only term in eq 4 that accounts for differences in size and shape of the involved molecules. Therefore, ln *γ*_*i*_^comb^ and the specific relation employed to determine it becomes of greater importance for polymer systems, such as ASD, than for conventional systems with low-*M* components only. The established SG combinatorial term was designed specifically for mixtures of small to moderate-sized molecules. Therefore, in case of polymers, it is generally recommended to replace SG by an alternative term that accounts not only for the combinatorial contribution, but also for free volume (FV) effects. These effects are recognized to play a significant role in polymer solutions and influence their thermodynamic behavior.^60,61,88−90^ Polymers and low-*M* compounds may significantly differ in the amount of FV in their pure states. Specifically, low-*M* solvents are usually more expanded than polymers, resulting in their bulk volume having a higher percentage of FV.^61,88^ Therefore, it can then be very important to explicitly account in a model for the overall change of the FV upon mixing. Accordingly, the activity coefficient equation reads
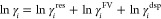
6
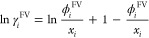
7where *x*_*i*_ is the mole fraction and φ_*i*_^FV^ is the free volume fraction of the component *i*.

Eq 7 has the same mathematical form as the Flory–Huggins combinatorial term, but replaces the classic volume fraction ϕ_*i*_ with the free volume fraction φ_*i*_^FV^
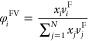
8Therein, *v*_*i*_^F^ denotes the free molar volume of component *i*
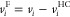
9where *v*_*i*_ is the liquid molar volume, while *v*_*i*_^HC^ is the molar hard core volume of the pure component *i*. The values for *v* and *v*^HC^ used in the present COSMO-SAC calculations are provided in Table S3. For *v* of both API and polymers, experimental values or, if unavailable, values from estimation methods were used (see Table S3 for details). Although *v* is, in principle, a function of temperature, the employed *v* values correspond to 298 K to keep the approach reasonably simple. This simplification can be justified by the fact that it is the difference between API and polymer in terms of their *v*^F^/*v* ratios that is the essential aspect in the FV approach, and this ratio can be considered as temperature-independent if both *v*_API_ and *v*_poly_ vary with *T* in a similar way.^61^Figure S2 illustrates that this appears to be a reasonable assumption. For each molecular species, the hard core volume *v*^HC^ was considered to be the van der Waals volume calculated on the basis of Bondi radii of individual atoms.^91^ This approach follows the methodology previously applied in the context of COSMO-SAC by Kuo et al.^60^ Specifically, *v*^HC^ values based on the Bondi atomic radii were determined using the fast calculation method proposed by Zhao et al.^92^

Note that the application of COSMO-SAC with the FV term requires two additional input parameters per molecular species: *v* and *v*^HC^. This situation slightly spoils the narrative of COSMO-SAC to require only molecular structure as an input and introduces additional parametrization cost. However, *v*^HC^ can easily be obtained from established Bondi radii. The determination of the liquid molar volume *v* (or density) generally represents a more serious challenge than that of *v*^HC^. If experimental *v* values are unavailable or their use is not preferred for the sake of maintaining a more predictive approach, they can be determined with estimation methods^93,94^ that, in principle, only require SMILES strings from the user.

#### 2.2.3 Model Configurations

Given the multiplicity of elements within the model, establishing a precise and standardized nomenclature is essential. The foundational COSMO-SAC model, denoted in this work simply as “CS,” exclusively encompasses the residual activity coefficient, with the combinatorial and dispersion terms omitted. Inclusion of any supplementary terms is systematically indicated through appropriate sub- or superscripts. For instance, the incorporation of the dispersion term is signified as CS_dsp_, while the integration of the combinatorial term is indicated as CS^SG^ or CS^FV^, corresponding to the SG or FV terms, respectively. Table 2 outlines all six distinct model configurations that arise from these variations, four of which are discussed in this work. The CS_dsp_^FV^ configuration was established as the benchmark model for comparison against two alternative models, namely CS_dsp_^SG^ and CS^FV^. The fourth configuration, CS_dsp_, was also explored, with its numerical results provided as part of COSMOPharm (see Section 3.4).

**Table 2:** Overview of the Possible COSMO-SAC (CS) Model Configurations

COSMO-SAC	Activity coefficient		
Notation^a^	residual	combinatorial	dispersion
CS	ln *γ*_*i*_^res^	–	–
CS^SG^	ln *γ*_*i*_^res^	ln *γ*_*i*_^SG^	–
CS^FV^	ln *γ*_*i*_^res^	ln *γ*_*i*_^FV^	–
CS_dsp_	ln *γ*_*i*_^res^	–	ln *γ*_*i*_^dsp^
CS_dsp_^SG^	ln *γ*_*i*_^res^	ln *γ*_*i*_^SG^	ln *γ*_*i*_^dsp^
CS_dsp_^FV^	ln *γ*_*i*_^res^	ln *γ*_*i*_^FV^	ln *γ*_*i*_^dsp^

a The reference model CS_dsp_^FV^ and two alternative models (CS_dsp_^SG^ and CS^FV^) are presented in the main text. Results for CS_dsp_ are only included in COSMOPharm.

### 2.3 σ-Profiles Used for API and Polymers

All employed σ-profiles were determined in this work, i.e., no database of predetermined profiles was utilized. This allowed for full control of the calculation details, including molecular geometries at which the QM calculations of σ, *A*, and, hence, the σ-profile were performed.

The σ-profiles for all API considered in this work were obtained from σ and *A* calculated using the Gaussian 16 software (revision C.01)^95^ considering the BVP86 density functional within DFT and the TZVP (triple-zeta valence polarized) basis set. The solvation was accounted for by using the conductor-like polarizable continuum model (CPCM)^96^ with infinite dielectric limit (i.e., keyword SCRF = COSMORS in Gaussian), which is an implicit solvation theory, considering the solvent to be a continuum field (therefore, no specific solvent selection is made). These single-point DFT/CPCM calculations typically take from a few to several tens of CPU minutes, depending on the API complexity (for example, GSF with 353 g mol^–1^ took 20 CPU minutes on an AMD EPYC 7543 2.80 GHz machine), and can be efficiently parallelized. For the five API, namely GSF, IBP, IMC, NPX, and PCM, the molecular geometries at which the DFT/CPCM calculations were performed correspond to the lowest-energy conformers in vacuum, and were determined in preceding work.^17^ Accordingly, the most stable gas-phase geometries of NIF and SIM were determined in this work. For generating the σ-profiles of the API from Gaussian COSMO files, we used the to_sigma.py script distributed with the employed COSMO-SAC package.^50^ The obtained geometries and σ-profiles are depicted in Figure S3 and provided numerically in COSMOPharm.

In principle, the molecular geometry has an effect on the calculated QM data. However, it was demonstrated^17,56^ that the specific molecular conformer used in the σ-profile calculation has a significant effect only in the case of API that can form intramolecular HB (intra-HB), while it is more or less negligible in case of API without this ability. The only API among the seven considered in this work that can form intra-HB is SIM. However, because (a) it was suggested that the specific intra-HB conformer is less stable than the non-intra-HB ones^78^ and (b) SIM is included in only two of the 35 binary systems, we did not study the effect of different API conformers on the COSMO-SAC results in this work.

**Figure 2:**
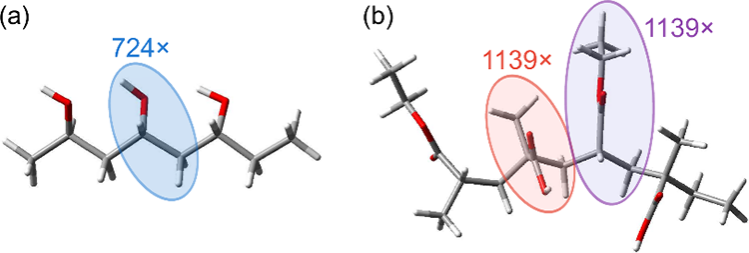
Schematic illustration of how oligomers were used to produce the σ-profile of polymers: (a) a trimer of the homopolymer PVA (32,000 g mol^–1^, *N*_units_ = 726) and (b) a tetramer of the copolymer EUD (212,000 g mol^–1^, *N*_units,A_ = *N*_units,B_ = 1140). The structures were drawn by GaussView^98^ and post-processed in Inkscape.^99^

The situation with respect to σ-profiles of polymers is generally more challenging and requires special treatment. It is too costly, impractical, or even impossible in terms of computational time to perform the required QM calculations for the entire polymer molecule with its true chain length, especially in case of high polymerization degrees (the computational cost of DFT calculations typically increases with the third to fourth power of the number of atoms). To rationalize the procedure for determining σ-profiles of polymers, a “replication” approach has been proposed by Kuo et al.^60^ that utilizes the fact that macromolecules are composed of many repeating units of one or more types. In this approach, QM calculations are performed only for a shorter oligomer molecule from which the σ-profile of a polymer molecule of any chain length can be determined by replicating the properties (charge density and surface area) of selected monomer unit(s) *n* times, where *n* is an integer ensuring that the virtual macromolecule has the targeted number of units *N*_units_. Regarding the oligomers, trimer and tetramer molecules are most often used for homopolymers and copolymers, respectively,^60,61,97^ but longer oligomers can also be used.^62^ For instance, when the σ-profile of a homopolymer with a total of *N*_units_ monomer units is to be determined, the central unit of a respective trimer is replicated (*N*_units_ – 2) times, because the two edge units are already included, as illustrated in Figure 2a. The replication approach assumes the central unit to be a representative average monomer whose properties propagate to the resulting σ-profile of the entire polymer as it undergoes replication. The identification of the unit(s) to replicate is therefore one of the key aspects of this approach. Although it represents a simplification and introduces some degrees of freedom to the technical details, it can be considered as established because it has been successfully used in previous works.^60−62^

Following ref (60), we used trimers for the considered homopolymers, namely, PDL, PVA, and the two PVP. For the copolymers EUD, PLGA50, PLGA75, and PVPVAc64, tetramers were employed. The termini of these short oligomers were capped by appropriate end groups to saturate chemical bonds and imitate the continuation of the chain. Specifically, the methyl group (CH_3_) was used in most cases. Only for one of the termini of PDL and the two PLGA, the methoxy group (CH_3_O) was considered.^19^ In the case of the copolymers, the alternating A-B-A-B arrangement of the two different units A and B within a tetramer molecule was considered. Syndiotactic oligomers (i.e., the pendant groups have alternate positions along the polymer backbone) were considered for all polymers, except PVA, for which an atactic trimer (i.e., pendant hydroxyl groups have random positions along the backbone) was used to reflect the fact that commercial PVA samples often contain atactic PVA chains.^100,101^ Then, to determine the optimal gas-phase conformers, we applied a strategy similar to that used for API. First, conformational analysis was performed at an MM level using the tool RDKit,^102,103^ and an appropriate low-energy structure was then refined quantum-mechanically with Gaussian at the BVP86/TZVP level of theory (all in vacuum). The final CPCM calculation at the same QM level was carried out to produce σ.

After the QM calculation of σ and subsequent σ averaging (see Section 2.2.1), the properties pertaining to segments of the central monomer unit(s) were replicated. For the homopolymers, the central unit of a trimer was replicated (*N*_units_ – 2) times, as described above. Accordingly, in the case of the copolymers, the central units A and B of tetramers were replicated (*N*_units,A_ – 1) times and (*N*_units,B_ – 1) times,^60^ respectively, as illustrated in Figure 2b. The values of *N*_units_, *N*_units,A_, and *N*_units,B_ for each polymer were determined from the reported molar mass and copolymer composition as provided in Table S2. The replication approach allows for the estimation of the σ-profile of a polymer chain of any length and, in the case of copolymers, of any mutual ratio of the different units just based on a single QM calculation of the respective oligomer. We took advantage of this principle in the case of the PVP homopolymers and PLGA copolymers.

The obtained σ-profiles for both oligomers and polymers, together with the considered geometries of the oligomers, are included in COSMOPharm and depicted in Figure S4. Also provided is a modification of to_sigma.py named to_sigma_poly.py, which allows for the determination of the σ-profile of a polymer based on an oligomer via the replication approach.

For completeness, the molecular surface area and volume of a polymer needed in the combinatorial SG term (together with those of API), were also determined from those of oligomer molecules calculated together with σ via QM, following the replication approach. The respective equations can be found in ref (60).

As there are relatively many degrees of freedom in the determination of polymer σ-profiles based on oligomer molecules, e.g., their length, tacticity, etc.,^60−62,97^ we examine in Section 3.3.3 how sensitive the COSMO-SAC predictions are to some methodological details.

### 2.4 Experimental Data and Statistical Analysis

To evaluate the predictive performance of COSMO-SAC for ASD, we used reliable and consistent experimental API solubility data. These data were provided by Mathers and co-workers^40,65−67,76,104−106^ and obtained using differential scanning calorimetry (DSC).^30,104^ An overview of all considered API–polymer systems and the individual experimental solubility data sources is given in Table 3.

**Table 3:** Overview of the API–Polymer Systems Considered in This Work and Sources of Their Experimental Solubility Data

Polymer/API	GSF	IBP	IMC	NIF	NPX	PCM	SIM
EUD		(65)	(106)		(106)	(106)	
PDL							(106)
PLGA50		(40)	(40)		(40)	(40)	(76)
PLGA75		(40)	(40)		(40)	(40)	
PVA		(66)	(66)		(66)		
PVPK12	(67)	(107)	(104)	(67)	(67)	(107)	
PVPK25	(67)			(67)	(67)		
PVPK30	(67)			(67)	(67)	(106)	
PVPVAc64	(67)		(107)	(67)	(67)	(106)	

The accuracy of COSMO-SAC solubility predictions was quantified based on the calculated deviation between the experimental values (indicated by the superscript “exp”) and those calculated using COSMO-SAC (“calc”)
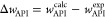
10This evaluation employed the average absolute deviation (AAD) and the average deviation (AD) as key metrics
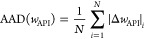
11
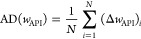
12where *N* is the number of experimental data points and *i* denotes an individual data point. Note that AAD primarily serves to quantify the prediction error, while AD offers additional insights. Specifically, the absence of an absolute value in AD allows for the determination of whether the COSMO-SAC model has a tendency to under- or overestimate the experimental data. Further, the average absolute relative deviation (AARD) was considered because of its routine usage in the ASD-oriented literature^10,32,38,41,67^
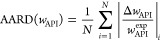
13

However, AARD requires careful assessment in the context of weight fractions. This is due to its inherent bias toward smaller values of *w*_API_ → 0. Therefore, the AARD values of the prediction results obtained in this study are only provided in COSMOPharm (see Section 3.4).

It is important to note that, in contrast to the *solubility* of API in polymers (SLE), there is a lack of experimental data on the *miscibility* of API and polymers (AAPS). The absence of such data is mainly attributed to the inherent challenges associated with AAPS measurements in ASD, primarily arising from the high viscosity of these systems. Consequently, alternative experimental LLE approaches are necessary. There are various techniques to characterize the miscibility of polymer systems, such as cloud-point measurements, DSC, X-ray powder diffraction, Raman mapping, and atomic-force microscopy.^6,108^ Methods with high spatial resolution, which enhance insight into API–polymer mixing on the molecular-to-nanometer scale, are also employed.^109−111^ In this work, we used AAPS information from Mathers et al.^40,65,66,104,105,107^ based on the DSC measurements of *T*_g_ of an API–polymer system as a function of temperature and composition. For a more detailed description of this methodology, see ref (40). However, the outcomes of these methods do not yield a continuous binodal curve. Instead, they produce a rather coarse network of temperature–composition points, providing qualitative indications of the AAPS boundaries without the exact composition of the two coexisting phases. As a result, any comparison between AAPS predictions from COSMO-SAC and experimental information can only be made qualitatively (e.g., in a yes-or-no manner, as done in this work). Furthermore, the experimental approaches to AAPS, unlike those to solubility, typically do not clarify the thermodynamic nature of the system, i.e., whether the observed (im)miscibility corresponds to thermodynamic equilibrium or is metastable or unstable.^6^ Therefore, since COSMO-SAC predicts the *equilibrium* AAPS based on the isoactivity condition (eq 3), a comparison of experimental and predicted AAPS should be made with sufficient caution.

## 3 Results and Discussion

The performance of COSMO-SAC for ASD is systematically presented, evaluated, and discussed here with respect to quantitative (numerical precision) and qualitative (AAPS prediction and polymer ranking) aspects. In Sections 3.1 and 3.2, only the results obtained from our “reference” modeling approach are considered, which is the CS_dsp_^FV^ configuration of COSMO-SAC, representing the fully equipped state-of-the-art COSMO-SAC-dsp model with the FV combinatorial term (see Section 2.2.3), together with the σ-profiles of API and polymers determined as described in Section 2.3. In Section 3.3, we inspect the sensitivity of the COSMO-SAC predictions to variations of different modeling aspects that represent possible departures from the reference approach. All numerical results obtained in this work, i.e., the calculated values of solubility (*w*_API_) and miscibility (*w*_API_^L1^, *w*_API_^L2^) and the corresponding deviations, are provided in the form of spreadsheets as part of COSMOPharm (cf. Section 3.4).

### 3.1 Quantitative Performance

**Figure 3:**
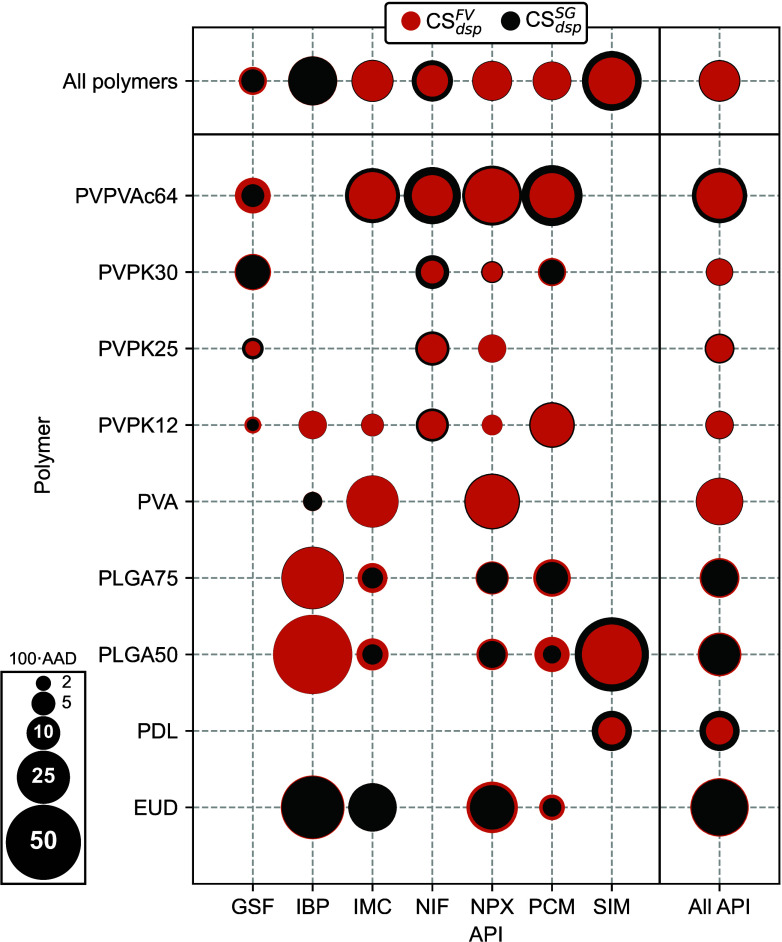
Graphical overview of AAD(*w*_API_) values derived from CS_dsp_^FV^ and CS_dsp_^SG^.

**Figure 4:**
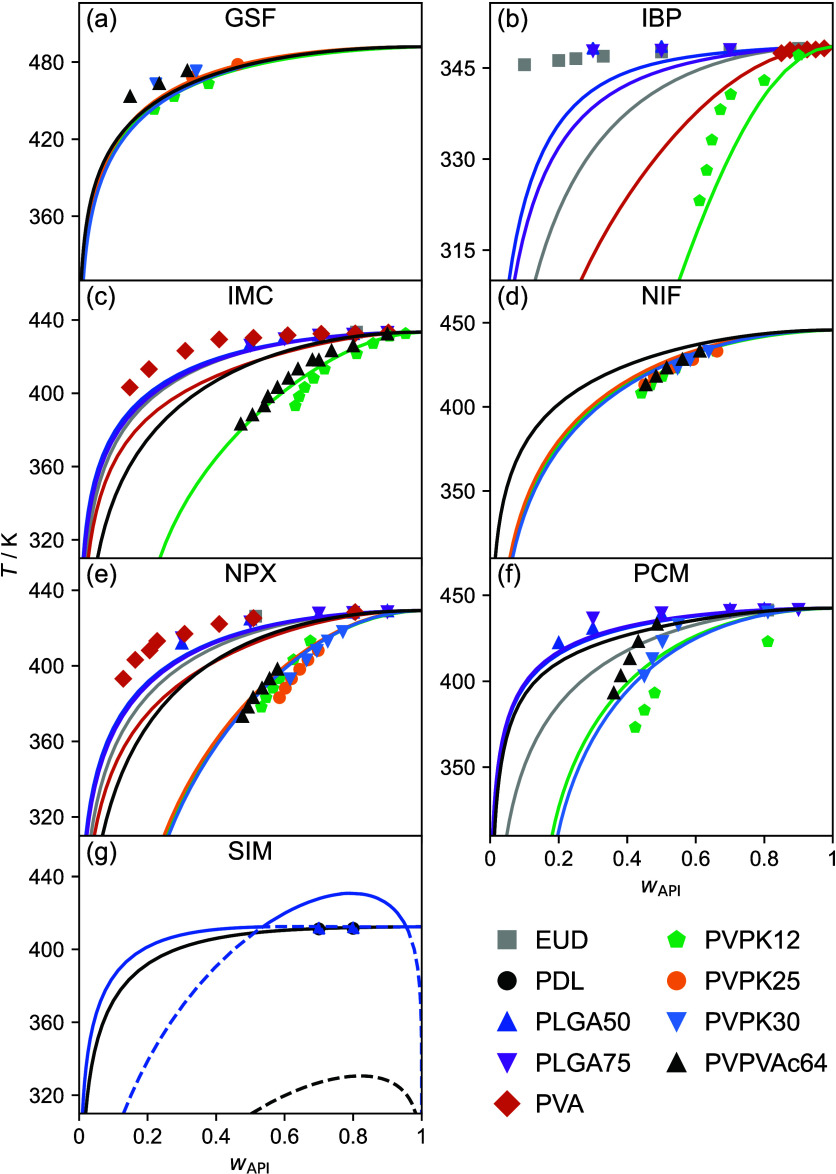
Solubility curves (solid lines) and AAPS curves (dashed lines) predicted by CS_dsp_^FV^ in comparison with experimental solubility data (symbols).

A visual overview of the AAD(*w*_API_) values from CS_dsp_^FV^ for each API–polymer system is presented in Figure 3. Therein, the columns and rows correspond to individual API and polymers, respectively. The row labeled “All polymers” aggregates the total AAD values for each API across all polymers, while the column “All API” performs an analogous aggregation for each polymer across all API. The “All polymers” and “All API” data, together with the corresponding AD(*w*_API_) values, are depicted in Figure S5. Phase diagrams featuring calculated solubility and AAPS curves are shown in Figure 4.

#### 3.1.1 Overall Performance

The total AAD(*w*_API_) value obtained from CS_dsp_^FV^ for all systems was 12.6%. For the sake of comparability with previous studies regarding predictions for ASD that primarily used AARD (eq 13), we add that the corresponding total AARD(*w*_API_) value is 33.3%.

**Figure 5:**
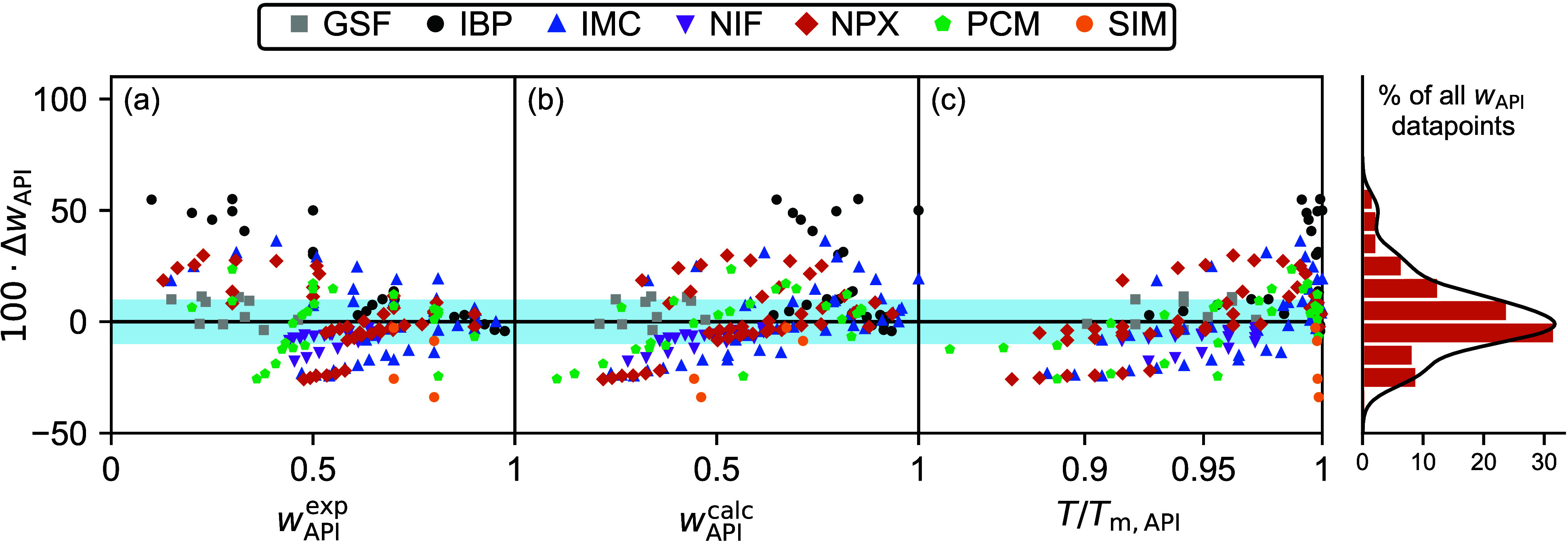
Δ*w*_API_ values of the individual data points vs (a) experimental *w*_API_ values, (b) predicted *w*_API_ values, and (c) *T*/*T*_m,API_ values, all obtained from CS_dsp_^FV^. Adjacent to the figures, an integrated density chart, complemented by a histogram, visually represents the distribution of Δ*w*_API_ values along the shared vertical axis. The shaded area denotes a Δ*w*_API_ range of ±10% in which about 60% of all data points reside.

The corresponding *median* of all  values is 8.5%, which indicates that the total AAD(*w*_API_) value is influenced by a smaller number of very high error values. The calculated error Δ*w*_API_ of the individual data points as a function of the experimental *w*_API_ values, the predicted *w*_API_ values, and the *T*/*T*_m,API_ values are shown in Figure 5, together with a histogram of the error values. All these visualizations support the above thesis, i.e., that the majority of the predicted data (almost 60%) fall within a relatively narrow error interval of Δ*w*_API_ = ±10%, and that there are relatively few data points with Δ*w*_API_ exceeding tens of percent, which negatively affects the total AAD value.

Building on the analysis presented in the preceding paragraph, there are notable differences in the performance of CS_dsp_^FV^ across individual systems. The accuracy is high for many systems, e.g., GSF–PVPK25, IBP–PVA, and NPX–PVPK30 with AAD values below 3% (the very lowest AAD values obtained approach the upper limit of the experimental uncertainty^67^). However, the accuracy is significantly worse for some other systems, e.g., IBP–EUD and IBP–PLGA50 with AAD of 34% and 53%, respectively. Among these latter systems, some individual data points show even larger deviations, though none exceed a Δ*w*_API_ value of 55%.

#### 3.1.2 Performance with Respect to API

We further inspect the performance of CS_dsp_^FV^ for individual API and polymers. Notably, the weakest performance was observed for IBP, with a total AAD of 20%. As delineated in Figure 3, the large total error predominantly stems from the IBP systems with EUD and PLGA. This suggests a challenge for CS_dsp_^FV^ in *numerically* capturing the reduced experimental compatibility observed in these systems (see Figure 4), as further evidenced by the experimentally detected AAPS in the IBP systems with the PLGA copolymers.^40,112^ However, as will be discussed in Section 3.2.1, CS_dsp_^FV^ does *qualitatively* predict AAPS and the overall limited compatibility of these systems. Interestingly, similar challenges regarding the numerical accuracy were recently encountered with PC-SAFT,^41^ indicating that the systems of IBP with EUD and PLGA pose a broader challenge for thermodynamic models. Conversely, most other API exhibited AAD values ranging from 8% to 13%. The most accurate results were found for GSF, with an overall error of just 6%. However, it should be acknowledged that the experimental dataset for GSF is limited to four systems, excluding those with EUD, PVA, or PLGA, that is, the systems for which some of the largest individual errors have been observed.

#### 3.1.3 Performance with Respect to Polymers

Among the polymers, EUD and PVA exhibited the largest total AAD values. Kuo et al.,^60^ applying COSMO-SAC with the FV term to VLE, also reported that systems containing PVA are not as well covered as mixtures with other polymers. Interestingly, both EUD and PVA are the only self-associating polymers included in this study, which means that they have both HB acceptor and donor sites within their monomer units, as detailed in Figure S4 (EUD possesses carboxyl groups, while PVA has hydroxyl groups; the other polymers have only HB acceptors). This characteristic, enabling these strongly interacting polymers to form both inter- and intra-HB,^113^ renders their modeling particularly challenging. Although COSMO-SAC effectively maps relevant short- and medium-range interactions, including HB,^50^ the high variability in possible HB patterns in these systems presents additional requirements that may exceed its current capabilities. Nevertheless, among EUD- and PVA-based systems, there are exceptions, like PCM–EUD and IBP–PVA, with better AAD values.

The second largest AAD was observed for PVPVAc64, with no significant outliers, unlike EUD and PVA, indicating a consistent performance across PVPVAc64-based systems (see Figure 3). The best results were achieved for the three PVP polymers, each showing an AAD of about 5%, a finding underscored by the fact that these results come from 13 PVP-containing systems, constituting about a third of all studied mixtures. Notably, only one of these (GSF–PVPK30) deviated significantly from the average AAD of about 5%. PDL also showed a comparable performance (6%), but the weight of this result is not as significant as that of the PVP polymers, since it is based on a single system.

The API solubility data and the corresponding errors predicted by CS_dsp_^FV^ for different polymers within the same group, i.e., (PVPK12, PVPK25, PVPK30) and (PLGA50, PLGA75), are very similar, as can be seen in Figure 4. This suggests that the polymer chain length *M* and copolymer composition hardly affect the SLE prediction results, which has already been reported in the context of PC-SAFT calculations.^112^

#### 3.1.4 Performance with Respect to All Data Points

Overall, CS_dsp_^FV^ tends to overestimate the API solubility, as indicated by the positive total AD value across all 35 systems (+2.5%). However, this overestimation is not universal, being observed for only four of the seven API, 19 of the 35 mixtures, and in 50% of the data points at experimental temperatures (*T*_exp_). Consequently, generalizations about a systematic bias in estimating *w*_API_ are ambiguous. A clearer tendency toward overestimation is seen in systems involving GSF and IBP as API and EUD, PVA, and PLGA as polymers, as evidenced by their high positive AD values in Figure S5. Conversely, systems tending to underestimations are often those with PVP and PVPVAc64 as polymers.

Figure 5 provides further insights into the behavior of the Δ*w*_API_ values as predicted by CS_dsp_^FV^. First, there seems to be a tendency to overestimate the solubility values, particularly when the corresponding experimental *w*_API_ values are below approximately 0.3, cf. Figure 5a. This overestimation often reaches up to tens of weight percent. Beyond an experimental value of *w*_API_ > 0.3, no distinct trend is discernible. Second, predicted solubilities lower than approximately 0.5 tend to be underestimated compared to the experimental data, cf. Figure 5b. This trend is qualitatively mirrored in Figure 5c, reflecting a general pattern where predicted solubility decreases with falling temperature. Third, the most significant Δ*w*_API_ values and instances of overestimation are observed at temperatures exceeding approximately 0.92·*T*_m,API_, see Figure 5c.

For completeness, we also explored potential correlations between the prediction error derived from CS_dsp_^FV^ and specific substance descriptors, including *T*_m,API_, *M*_API_, and *M*_poly_. The results are presented and discussed in Section S5.1.

### 3.2 Qualitative Performance

This section provides an insight into qualitative aspects of the predictive performance of CS_dsp_^FV^ for ASD. In particular, the capability of CS_dsp_^FV^ to qualitatively predict the existence of AAPS and correctly rank the polymers based on their compatibility with the API is assessed.

#### 3.2.1 Prediction of AAPS

AAPS represents another important aspect with respect to the mutual compatibility of API and polymers.^6^ Previous experimental observations revealed the occurrence of AAPS in only four of the 35 studied systems: IBP–PLGA50, IBP–PLGA75, NPX–PLGA75, and NPX–PVA. For the remaining systems, AAPS has either not been observed in 25 cases or has not yet been investigated in 6 instances, as summarized in Table S4 in the SI. Note that three of the four systems that show AAPS contain one of the PLGA as the polymer.

**Figure 6:**
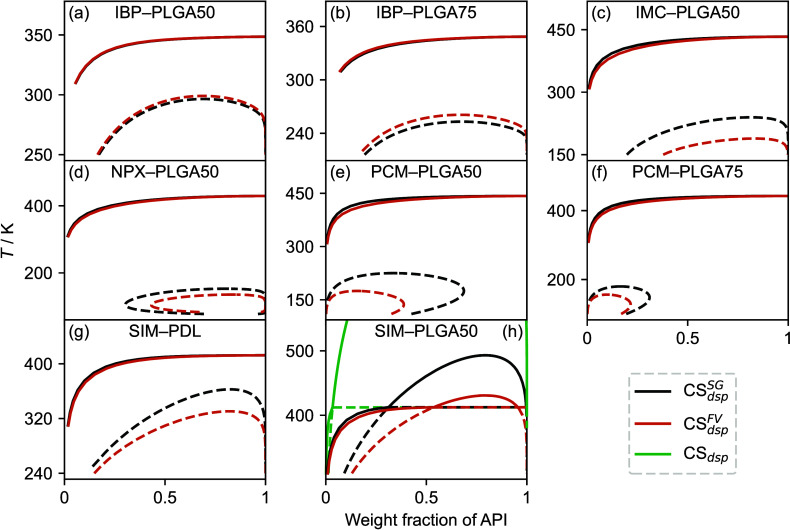
Phase diagrams over a broader temperature range for the systems predicted by CS_dsp_^FV^ and CS_dsp_^SG^ to exhibit AAPS (dashed lines). Note: All SLE curves (solid lines) obtained from CS_dsp_^FV^ are depicted in Figure 4 and are repeated here for completeness.

A thorough computational analysis of AAPS performed in this work reveals that CS_dsp_^FV^ does predict AAPS for eight of the 35 systems (see Figure 6). All of them contain either PLGA50, PLGA75, or PDL, i.e., polymers based on poly(lactic acid), cf. Table S2 for the composition of the polymers. This obvious tendency to predict AAPS for PLA-based systems can generally be considered in good qualitative accordance with experimental observations.^40,112^ More specifically, CS_dsp_^FV^ qualitatively correctly predicts the existence of AAPS in case of the IBP–PLGA50 and IBP–PLGA75 systems. Quantitatively, the predicted AAPS regions are underestimated; while CS_dsp_^FV^ predicts the corresponding upper critical solution temperature (UCST; see Section S2) to be in the range 250–300 K, the experiments reported AAPS to occur even at 420 K, which was the maximum temperature considered.^40^ However, a quantitative evaluation is not what this section aims at, because (a) AAPS is computationally very sensitive to the modeling approach and (b) precise experimental AAPS boundaries are not known. In this context, it is only important that CS_dsp_^FV^ does predict AAPS in these cases. For systems SIM–PDL and SIM–PLGA50, it cannot be evaluated whether the presence of an AAPS predicted by CS_dsp_^FV^ is correct or not, since these systems have not yet been studied experimentally in terms of AAPS. A similar situation of unavailable experimental information also applies to four other systems (see Table S4), but CS_dsp_^FV^ does not predict AAPS for them.

As for the other four systems for which CS_dsp_^FV^ does predict AAPS (IMC, NPX, and PCM with PLGA50, and PCM–PLGA75), none of them has been experimentally found to show AAPS. However, it is important to note that the AAPS regions of these four systems were predicted to exist at very low temperatures below 200 K, while ASD experiments typically do not consider temperatures lower than 298 K. Furthermore, even if these systems do really show a tendency to form AAPS, its formation is expected to be kinetically very much hindered due to the temperature below *T*_g_ (as explained in Section S2). These circumstances would make the observation of AAPS nearly impossible within a sensible experimentation time frame.

Another interesting aspect is the closed-loop AAPS behavior predicted for some of the systems. Such an LLE type exhibits not only a UCST, but also a lower critical solution temperature (LCST), with UCST > LCST, and is not abnormal in the context of polymer systems, especially those with hydrogen bonding.^68,114^ The closed-loop AAPS for the mentioned systems is found at low temperatures and, in case of PCM–PLGA50 and PCM–PLGA75, at relatively low API concentrations. It is remarkable that a similar AAPS behavior in these two PCM-based systems was entirely independently predicted with PC-SAFT.^41^

The only systems with an experimentally observed AAPS that CS_dsp_^FV^ found to be homogeneous are NPX–PVA and NPX–PLGA75. However, while CS_dsp_^FV^ predicts AAPS for PLGA50, but not for PLGA75, the experimental results indicate the opposite. This could still serve as a very rough indicator of the potential risk of AAPS when NPX is mixed with PLGA-type polymers.

For all systems, except for SIM–PLGA50, the predicted AAPS region lies completely below the API solubility curve, which represents a thermodynamically metastable AAPS behavior. From the perspective of formulation design, the occurrence of metastable AAPS behavior is considered as an important (yet unfavorable) aspect, along with the potential of a model to correctly predict its existence or absence for a given system. In this context, when screening for AAPS, we should not restrict ourselves to situations where AAPS has been experimentally observed and to question of whether the equilibrium predictions from CS_dsp_^FV^ align with these observations. Equally important are systems not showing AAPS and the corresponding potential of CS_dsp_^FV^ to reflect this phase behavior. In this regard, it can be noted that CS_dsp_^FV^ correctly predicts the absence of AAPS in 21 of the 35 systems considered, including all systems with GSF and NIF as the API, and PVP and PVPVAc64 as the polymer, as detailed in Table S4.

As a final remark regarding AAPS, note that the decreasing ratio of the PLA units in the polymer molecule (i.e., PDL > PLGA75 > PLGA50) obviously decreases the API–polymer miscibility predicted by CS_dsp_^FV^. In other words, it increases the extent of the predicted AAPS region. This illustrates the elevated sensitivity of the LLE results to modeling details, such as copolymer composition, compared to that of SLE. It also suggests that CS_dsp_^FV^ can, in principle, captures these nuances.

#### 3.2.2 Polymer Ranking

As discussed in Section 3.1, the results from CS_dsp_^FV^ for ASD may achieve various degrees of numerical accuracy, ranging from AAD(*w*_API_) values of only a few percent to 50%, depending on particular systems. However, what typically makes COSMO-type models outstanding is their qualitative performance for screening purposes. In the context of pharmaceutical applications, solvent screening for drugs is of great importance, where the predictions are used to qualitatively rank a large set of solvents with respect to their compatibility with a given API. At the same time, the numerical (dis)agreement of predicted and experimental values is typically irrelevant. The power of COSMO-based models in solvent screening for drugs has been tested and proved in several studies, but with a focus on low-*M* solvents^17,51,55^ while polymers were not regarded. Therefore, the efficacy of COSMO-SAC for qualitative “polymer” screening in the context of ASD remains to be explored in this work, and the findings are outlined in the following.

**Figure 7:**
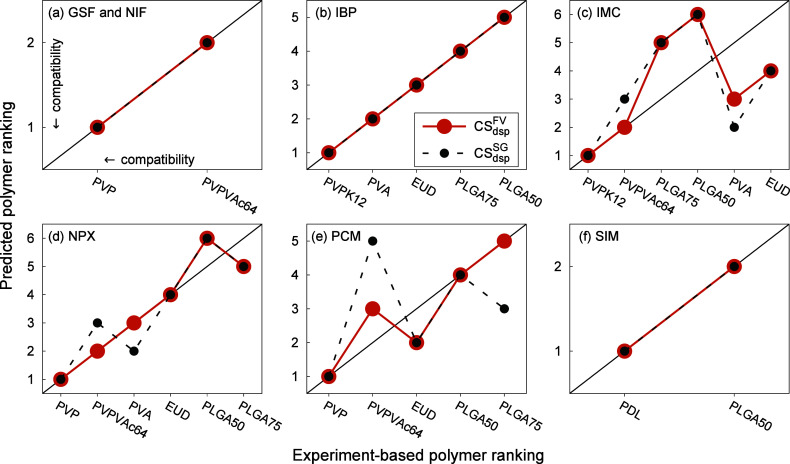
Ranking of the polymers based on their compatibility with the API as predicted by CS_dsp_^FV^ and CS_dsp_^SG^ compared to the experiment-based polymer ranking. For each API, the axes depict the experiment-based and predicted rankings, respectively, arranged from the most to the least compatible polymer in sequential order. The compatibility thus increases from right to left and from top to bottom, as illustrated in figure (a). The lines serve as a guide to the eye.

For each API, we ordered the polymers with respect to their compatibility with the API as predicted by CS_dsp_^FV^ and compared that to the experiment-based order. Specifically, both the predicted and experiment-based orders were determined from API solubility values in polymers and the absence or existence of AAPS (closer details on this procedure and explanation of the term “experiment-based order” are provided in Section S7). The results of this polymer ranking analysis are shown in Figure 7 by means of parity plots. For this purpose, the three PVP polymers (that only differ in their chain length) were grouped together as “PVP”. This treatment was motivated by the close proximity of the solubility lines for a given API in these three PVP. These lines typically form a “bundle” of curves that are challenging to distinguish, a characteristic often observed in both predicted and experimental data.

As can be seen in Figure 7, for GSF, NIF, IBP, and SIM, CS_dsp_^FV^ provides a perfect polymer ranking in comparison with the experiment-based one, which is indicated by the fact that the polymer order points lie on the diagonal of the graphs. In these cases, such a performance means that no additional experimental measurements would be needed to refine the predicted polymer order. While the perfect polymer ranking is not surprising in the case of GSF and NIF, for which CS_dsp_^FV^ showed the best quantitative performance, it can be considered remarkable for IBP and SIM, where the poorest numerical accuracy was achieved. This demonstrates that even numerically inaccurate results may be valuable when used qualitatively for polymer ranking.

While the predicted polymer order for the other three API was not entirely correct, the inaccuracies are limited to an incorrect order of two polymers or two pairs of polymers. For instance, EUD and PVPVAc64 were predicted to exhibit the second and third best compatibility with PCM, respectively, despite the experiment-based order showing the opposite (see Figure 7e). For IMC, CS_dsp_^FV^ swapped the order of the pairs (PLGA50, PLGA75) and (PVA, EUD), which spoils the corresponding parity plot in Figure 7c. However, the fact that these four polymers show significantly lower compatibility with IMC than PVPK12 and PVPVAc64 is reflected correctly. Since these inaccuracies differ from one API to another, there seems to be no systematic error in the ranking with respect to individual polymers.

As can be seen in Figure 7, certain polymer types consistently exhibit better compatibility with the API than others. Specifically, PVP polymers typically have the best compatibility with API, followed by PVPVAc64,^10,22,32,67^ while the biocompatible PLGA copolymers are reported to have relatively poor compatibility (though being sufficient for formulation purposes).^40,112^ These experimentally observed “behavioral” patterns of polymers appear to be captured very well by the predictions made by CS_dsp_^FV^. The model successfully predicts that PVP always exhibit the best compatibility with API, while PLGA tend to have the poorest (or one of the poorest), and PVPVAc64 falls in between, typically right after PVP. The accurate prediction of the relative order of PVP and PVPVAc64 by CS_dsp_^FV^ indicates that it can successfully anticipate the decrease of compatibility with API resulting from the chemical modification of PVP through the incorporation of PVAc units in the form of a diblock copolymer. It was recently demonstrated that the solubility of a given API in a polymer often correlates with the HB part of the API–polymer interaction energy (unless sterically hindered).^10^ That could indicate that also the variation of HB interaction strength between an API and different polymers is qualitatively captured by CS_dsp_^FV^. Regarding the detailed mutual order of PLGA50 and PLGA75, this is predicted correctly in three out of four cases (IBP, IMC, and PCM). The only instance where an incorrect prediction occurred is NPX, which is associated with the reverse prediction of AAPS for the two NPX–PLGA systems in comparison to the experiment, as discussed above.

### 3.3 Sensitivity Analysis

So far, the prediction results achieved with CS_dsp_^FV^ and a single σ-profile for each polymer (as described in Section 2.3) have been presented and discussed. Here, the sensitivity of the results to variations in both the configuration of the model and the σ-profiles of the polymers is inspected.

#### 3.3.1 Staverman–Guggenheim or Free Volume Term?

As mentioned in Section 2.2.2, when COSMO-type models are applied to systems containing polymers, it is generally recommended to replace the standard SG combinatorial term with the FV term due to its better performance. However, a detailed analysis of previously reported computational results^60,61^ reveals that this improvement is not automatic for each individual system. Rather, it is observed for the majority of them and for the total deviation calculated over all systems. Therefore, this section aims at an evaluation and quantification of what can be expected when replacing ln *γ*_*i*_^FV^ (CS_dsp_^FV^) with ln *γ*_*i*_^SG^ (CS_dsp_^SG^) in case of API–polymer systems.

**Figure 8:**
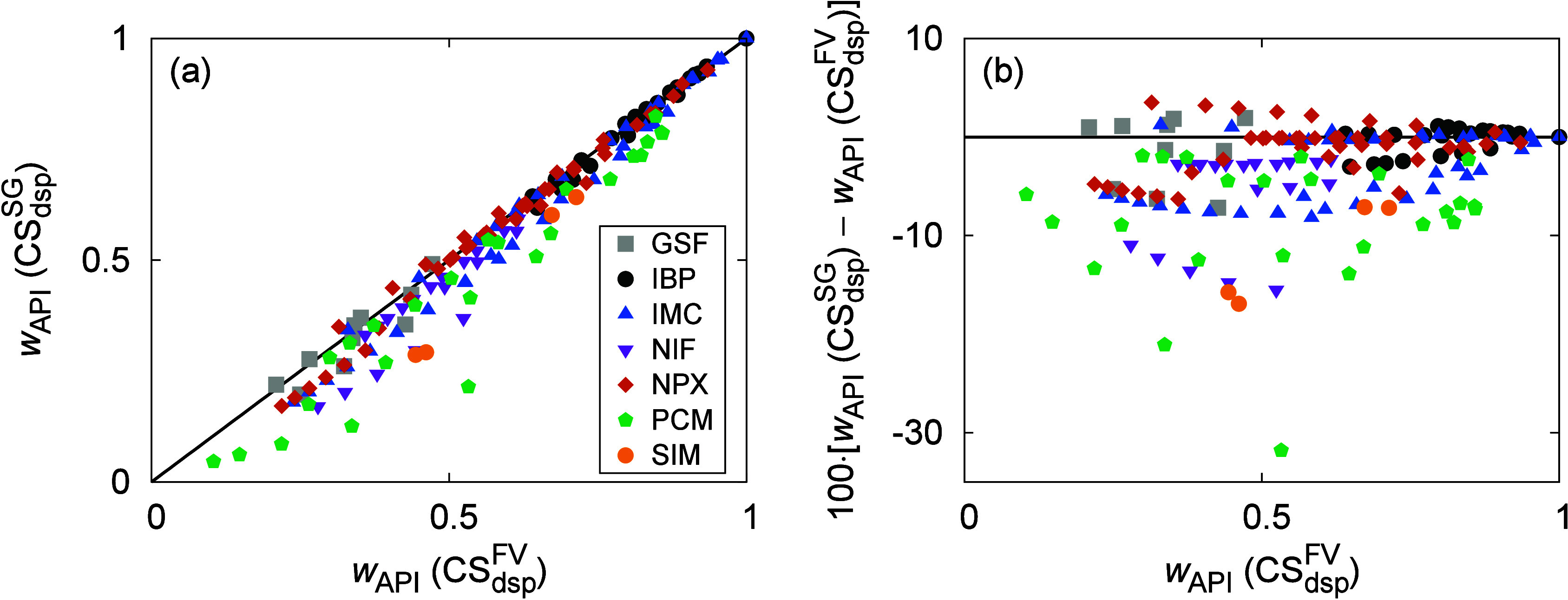
Sensitivity analysis regarding the combinatorial contributions in terms of the calculated API solubility values: (a) CS_dsp_^SG^ vs CS_dsp_^FV^ by means of a parity plot and (b) difference between CS_dsp_^SG^ and CS_dsp_^FV^ as a function of *w*_API_ (CS_dsp_^FV^). The data points were calculated at *T*_exp_.

A comparison of the API solubility values calculated by CS_dsp_^FV^ and CS_dsp_^SG^ is shown in Figure 8. First, it can be seen that the switch from CS_dsp_^FV^ to CS_dsp_^SG^ leads in most cases to a *decrease* of the predicted solubility values (for 26 of the 35 systems and 75% of the data points calculated at *T*_exp_). The systems that did not follow this prevailing trend and for which CS_dsp_^SG^ produced higher *w*_API_ values were often those with IBP and GSF as the API and PVA as the polymer (which is discussed in more detail below). An average difference between *w*_API_ values calculated by CS_dsp_^FV^ and CS_dsp_^SG^ at *T*_exp_ was approximately only −3%, with a maximum individual difference of −32% for the PCM–PVPVAc64 system. (Not surprisingly, the largest difference of the respective AAD values was also found for this system, as discussed below).

Another interesting aspect was that the difference between the *w*_API_ values from CS_dsp_^FV^ and CS_dsp_^SG^ appears to show a certain “parabolic” trend (toward both negative and positive values) with respect to the composition of the system, cf. Figure 8b. To validate this observation, we redrew Figure 8b, this time using data covering a wider *w*_API_ range instead of those at *T*_exp_ for a more complete picture. The results are shown in Figure S8a and confirm the hyberbola-like behavior. Specifically, the largest absolute differences between CS_dsp_^FV^ and CS_dsp_^SG^ appear to be located mostly around the middle of the *w*_API_ interval, whereas they gradually decrease to zero toward both edges of the composition interval. While going to zero when *w*_API_ → 1 is a consequence of the natural behavior of the activity coefficients (both ln *γ*_API_^FV^ and ln *γ*_API_^SG^ → 0 for *x*_API_ → 1), it is a bit surprising at the opposite edge of the *w*_API_ interval when *w*_API_ → 0. This suggests that, under these conditions, both combinatorial terms contribute similarly to ln γ_API_ at saturation, which is discussed in more detail in Section S8.1.

A qualitatively similar behavior is seen if *w*_API_ (CS_dsp_^FV^) is replaced with the corresponding *T*/*T*_m,API_ values on the horizontal axis, as shown in Figure S8b, since *w*_API_ generally decreases with temperature. As a result, the difference between the API solubilities calculated by CS_dsp_^FV^ and CS_dsp_^SG^ is small to negligible at lower *T*, corresponding to, e.g., the storage temperature.

With regard to the numerical agreement with experimental solubility data, switching from CS_dsp_^FV^ to CS_dsp_^SG^ slightly increases the total AAD(*w*_API_) value calculated over all 35 systems from 12.6% to 13.9%. Although this means that the application of the FV term has a positive effect on the overall accuracy for the considered API–polymer systems, the relatively small overall numerical difference still keeps CS_dsp_^FV^ comparable to CS_dsp_^SG^. However, some individual systems show more significant differences, as in the case of PCM–PVPVAc64 mentioned above, for which AAD values of 16% and 31% were obtained from CS_dsp_^FV^ and CS_dsp_^SG^, respectively. As can be seen in Figure 3, the difference in accuracy of the two model variants may generally manifest itself differently from one system to another. Specifically, there are systems for which they provide almost identical results (e.g., IBP–PLGA, IMC–PVPK12, and NPX–PVP), systems for which CS_dsp_^SG^ produces worse results (e.g., PCM–PVPVAc64, all NIF systems), and even systems for which CS_dsp_^SG^ outperforms CS_dsp_^FV^ (e.g., GSF–PVPVAc64, NPX–EUD, PCM–PLGA). Therefore, CS_dsp_^SG^ can have both a negative or a positive effect on the performance, depending on the individual system. For example, in cases where CS_dsp_^SG^ provides overestimated results, the application of CS_dsp_^FV^, with its prevailing tendency to indicate a better compatibility than CS_dsp_^SG^, cannot improve the accuracy. This observation is not surprising in the context of the results of similar studies,^60,61^ but rather underlines the fact that the improvement achieved with CS_dsp_^FV^ appears to be somewhat less systematic and automatic, although the prevailing trend across all systems is in favor of CS_dsp_^FV^, as it produces more accurate results than CS_dsp_^SG^ for 5 of the 7 API, 20 of the 35 systems, and 66% of the data points calculated at *T*_exp_. For comparison, Kuo et al.^60^ and Loschen and Klamt,^61^ for VLE of a low-*M* solutes in polymers, reported FV to outperform SG for 62% and 87% of the considered systems, respectively.

With regard to the qualitative performance, Figure 7 shows that CS_dsp_^SG^ outperforms CS_dsp_^FV^ in terms of the polymer ranking only for GSF among the API. For the remaining API, the ranking derived from CS_dsp_^SG^ is either identical (in case of GSF) or, more frequently, slightly less successful. Nevertheless, the overall qualitative outcome, highlighting PVP as having the best compatibility and PLGA as having the poorest, remains intact. Similarly, CS_dsp_^SG^ provides the same information as CS_dsp_^FV^ for AAPS, as can be seen in Figure 6. Quantitatively, CS_dsp_^SG^ tends to provide a slightly larger immiscibility than CS_dsp_^FV^ for 6 of the 8 systems for which AAPS was predicted, as measured by the higher UCST values in these cases. This, together with the observed decrease of API solubility, further underlines the overall tendency of CS_dsp_^SG^ to predict poorer API–polymer compatibility than CS_dsp_^FV^.

Although the change in FV in polymer–low-*M* solvent systems may have a considerable impact on their phase behavior,^88^ and its explicit inclusion may significantly improve the accuracy of COSMO-SAC for such systems,^60,61^ the results presented in this section show that the impact of the FV term on SLE/LLE of the API–polymer mixtures is less pronounced. The results suggest that the application of the FV term is beneficial, as it slightly improves the overall numerical accuracy, but it is not essential, as both CS_dsp_^FV^ and CS_dsp_^SG^ provide comparable qualitative and quantitative results. In other words, the absence of the FV term in a particular COSMO-SAC implementation or the unavailability of the volumetric data (*v* and *v*^HC^) required by FV do not pose significant numerical accuracy issues for COSMO-SAC in the context of ASD. Therefore, pharmaceutical formulators would not make a significant mistake if they use COSMO-SAC with the standard SG term, especially when the difference between CS_dsp_^FV^ and CS_dsp_^SG^ is practically zero at the storage temperature. The option to disregard the FV term also eliminates the necessity for both *v* and *v*^HC^. This means that COSMO-SAC can be used in completely QM-aided regime without any auxiliary input data (except the API fusion properties in case of SLE), which is represented by CS_dsp_^SG^. This reduces the parametrization costs in the overall performance–cost ratio.

**Figure 9:**
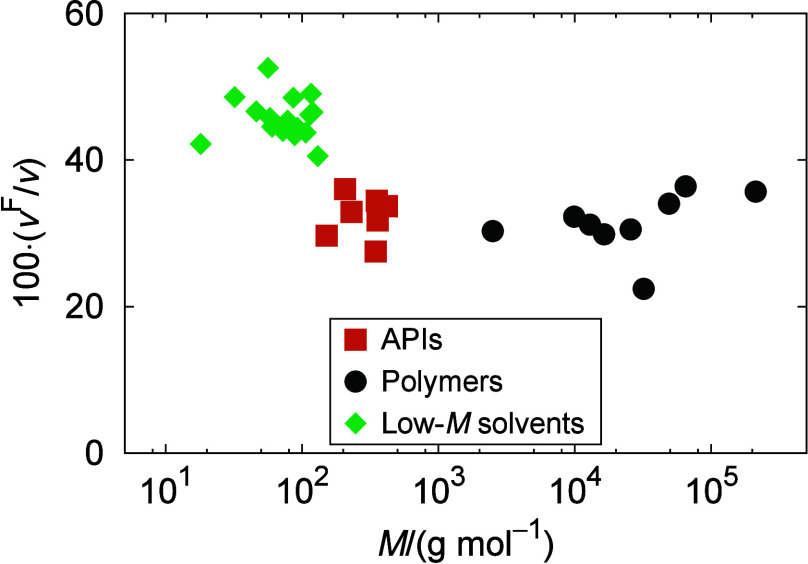
Ratio of FV to total bulk volume at 298 K of the API and polymers studied in this work, and a selection of ordinary low-*M* solvents considered in refs (60, 61).

Next, it is attempted to explain the observation that the present results do not show as significant an improvement through the application of the FV term as reported in other studies. The difference of the API activity coefficient, obtained from the FV term and a classical combinatorial term is given by^89^
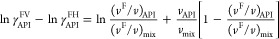
14In eq 14, *v*_mix_ = *∑*_*i*_*x*_*i*_*v*_*i*_, , and ln *γ*_API_^FH^ is the Flory–Huggins (FH) combinatorial term. Although the FH term is not exactly the SG term used in this work, for simplicity, we use it at this point for some general considerations (following the discussion given by Elbro et al.^89^). First, eq 14 indicates that the difference between the FV and FH terms is zero only when (a)  and (b) *x*_API_ → 1. Then, the larger the difference between  and , the more significant the FV contribution becomes. Therefore, we plotted the FV ratios of the pure API and polymers, and also selected low-*M* solutes considered in previous studies^60,61^ as a function of molar mass in Figure 9. The figure reveals that the FV ratios of low-*M* solvents are generally higher (46% on average) than those of the polymers (31%), while the FV ratios of the API (32%) are comparable to those of the polymers. Therefore, it is this relatively small difference of the FV ratios of the pure API and polymers, resulting in their mixing generally not causing a significant change of the FV, that could explain the overall low impact of the FV term observed in this study. These principles can also explain some other observations made in the above paragraphs. The respective discussion is provided in Section S8.2.

To complete the picture about the effect of the combinatorial terms of ln γ_*i*_, we also performed one additional run for all systems, but this time without any combinatorial contribution to ln γ*_i_* (i.e., neither FV nor SG). Such a model configuration, denoted CS_dsp_, only included the residual and dispersion terms and could not account for any effect stemming from the different molecular sizes. The total AAD obtained from CS_dsp_ was 26%, indicating an “entropic catastrophe” manifested by a significant decrease of accuracy compared to CS_dsp_^FV^ and CS_dsp_^SG^. Specifically, CS_dsp_ exerted a clear tendency to substantially underestimate the compatibility, as illustrated in Figure 6h (all data from CS_dsp_ can be found in COSMOPharm). Therefore, it can be concluded that it is critical to employ a combinatorial term when COSMO-SAC is applied to ASD, while its detailed form (FV or SG) appears to be less important.

#### 3.3.2 Role of the Dispersion Contribution

In this section, the effect of the dispersion contribution on the COSMO-SAC results is inspected and quantified for the considered API–polymer systems. To this end, all systems were recalculated once again, but this time the dispersion term in eq 4 was turned off (i.e., ln *γ*_*i*_^dsp^ = 0). This model is denoted CS^FV^. The results obtained from CS^FV^ were then compared with those of CS_dsp_^FV^.

It was found that the dispersion term has a negligible numerical effect on the results, which is manifested by the fact that both SLE and LLE curves calculated with CS^FV^ and CS_dsp_^FV^ are effectively identical. This is illustrated in Figure S11a which compares the solubility values predicted by CS_dsp_^FV^ and CS^FV^ by means of a parity plot. The average difference between *w*_API_ values from CS_dsp_^FV^ and CS^FV^ is about 1 ppm, with the maximum individual difference encountered for the GSF–PVPK12 system (0.2%). Similarly, the total AAD(*w*_API_) values were 12.6% for both CS_dsp_^FV^ and CS^FV^. It was also found that the negligible difference between CS^FV^ and CS_dsp_^FV^ applies even to LLE, which are otherwise more sensitive to the modeling approach. As a result, although the dispersion correction may have positive effect for VLE of ordinary low-*M* systems,^81^ it may be turned off without any notable effect on the COSMO-SAC results in case of API–polymer systems, which further (yet slightly) improves the performance–cost ratio. Therefore, the SLE and LLE results and the corresponding statistics for CS_dsp_^FV^ shown, e.g., in Figures 3, 4, 6, and 7 are also valid for CS^FV^, which explains why CS^FV^ is not depicted separately therein. In Section S9, we explore the difference between CS_dsp_^FV^ and CS^FV^ in more detail, concluding that a possible reformulation of the dispersion contribution by means of, e.g., surface area fractions of the components might be needed in case of polymer systems.

#### 3.3.3 Sensitivity to Polymer Treatment

In the preceding sections, the sensitivity of COSMO-SAC to variations regarding the combinatorial and dispersion contributions to ln γ_*i*_ was inspected. Now, it is focused on how the model reacts to changes in the σ-profiles of the polymers, which is directly related to the residual term.

As mentioned in Section 2.3, the determination of polymer σ-profiles has a large number of methodological degrees of freedom.^60−62^ The results presented so far indicate that there is certainly some room for improvement in numerical accuracy of the model. Therefore, this section inspects whether it is possible to achieve better results when longer or structurally different oligomer molecules are considered in the polymer σ-profile determination, in other words, when a different strategy to sample the representative monomer properties for replication is employed. All calculations were performed with the CS_dsp_^FV^ configuration.

To discuss the above question, we selected the PVP polymers and PVA. PVP were chosen because they are the most frequent polymeric component within the considered systems. PVA was chosen because its systems showed some of the poorest results and, at the same time, it has an interesting HB behavior.

##### PVA: Hydrogen Bonding and Structure Sensitivity

PVA, as a self-associating polymer, can form three types of HB through its hydroxyl groups: intermolecular API–PVA, PVA–PVA, and also intra-HB within a PVA macromolecule, which allows PVA to form strong HB networks.^113^ This significantly influences its thermodynamic behavior,^101,113,115^ including a relatively low compatibility with water or API.^66,114^ Mainly due to the intra-HB, the properties of PVA-based systems calculated with COSMO-type models can be affected by specific oligomer structures considered in the determination of σ-profile for PVA.^17,56^

First, it is focused on how sensitive the model is to the tacticity of PVA. In addition to the atactic trimer considered so far (*at*-3mer), we examined both a syndiotactic (*st*-3mer) and isotactic trimer (*it*-3mer). Geometries of these trimers and the corresponding σ-profiles of virtual PVA molecules of 32,000 g mol^–1^ are shown in Figure S13. Despite the different tacticities, all three trimers exert sequential intra-HB between the adjacent OH groups.

**Figure 10:**
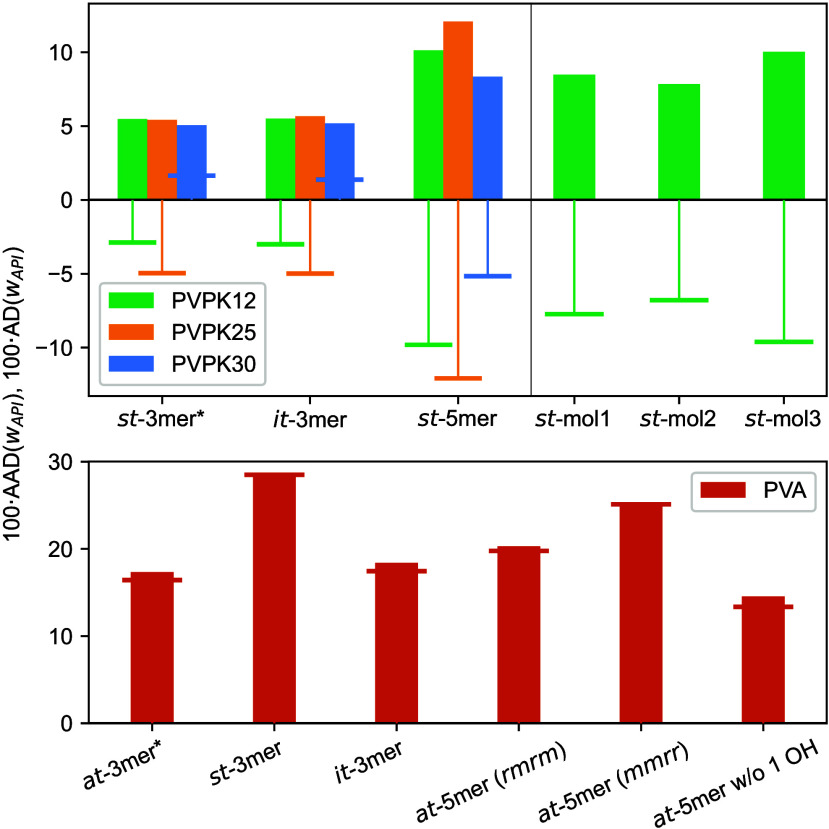
Sensitivity analysis regarding the polymer σ-profiles: total AAD and AD values for (top) PVP and (bottom) PVA calculated with different σ-profiles based on the different molecules. The asterisk (*) denotes the oligomer considered in the reference approach.

The results obtained with the σ-profiles of PVA based on the three trimers are shown in Figure 10 in terms of total AAD and AD. While both *at*-3mer and *it*-3mer provided comparable results, *st*-3mer produced higher *w*_API_ values, thus raising the observed overestimation and increasing the total error. A possible explanation is that, in a syndiotactic molecule, the HB sites are somewhat more accessible to the solute for interactions due to the generally greater distance between the adjacent OH groups compared to an atactic and, particularly, isotactic molecule. This explanation can be supported by the fact that the OH part of the σ-profile derived from *st*-3mer contains a HB-acceptor peak (around a σ value of 0.014 e Å^–2^) that is higher than that of the other PVA oligomers.

Another degree of freedom consists in the length of the oligomer. Regarding homopolymers, the use of trimers (with the central monomer being replicated, as applied in this study so far) is considered as a trade-off between accuracy and computational costs.^60^ To inspect this, we developed alternative σ-profiles of PVA based on pentamers instead of trimers. In addition to the oligomer length, another difference consisted in the fact that not only the single middle *unit* but the whole middle *triad* (i.e., the three central adjacent units) was used for the replication in the case of pentamers, as illustrated in Figure S13. This approach should theoretically lead to an improved sampling of the representative properties for the subsequent replication, as three units of a pentamer can cover more from the behavior of monomer units than a single unit of a trimer. Since the total of two meso (*m*) and two racemo (*r*) diads present in an *at*-5mer can be distributed differently, we included two different arrangements: *rmrm* and *mmrr*, as illustrated in Figure S13. Interestingly, Figure 10 shows that considering the *at*-5mers increased the predicted compatibility and, thus, led to even higher AAD values compared to the *at*-3mer, but still lower than the *st*-3mer.

The last test regarding PVA consisted in a modification of the HB ability of an oligomer. The API solubility can be related to the HB interaction energy, and the HB interaction energy is associated with the number of HB between an API and a polymer.^9,10^ Therefore, a reduction of the number of OH groups in PVA can, in principle, be expected to decrease the API solubility in it. This would have a positive effect for the accuracy, because all PVA σ-profiles considered so far led to overestimated solubilities. To examine this approach, we included a chemically modified *at*-5mer of PVA whose central triad lacked one OH group, cf. Figure S13 (“*at*-5mer w/o 1 OH”). Such a model molecule imitates a (hypothetical) situation that every third OH group in a virtual PVA chain is unavailable to the interaction with the API (e.g., either due to an intra-HB with a more distant fragment of the same molecule, intermolecular PVA–PVA HB, or structural irregularities^100^). Figure 10 reveals that the above expectations were relevant, as the modified *at*-5mer led to the lowest *w*_API_ values from the entire set of PVA oligomers, thus reducing the corresponding AAD. Although the reduced AAD was still comparable to that of the reference *at*-3mer, the chemical modification of the *at*-5mer qualitatively showed a possible route to customize the COSMO-SAC modeling approach with respect to the intricate HB behavior of polymers like PVA.

##### PVP: Tacticity and Conformational Effects

In addition to the reference *st*-3mer, we included an *it*-3mer to examine the effect of tacticity in the case of PVP. While the tacticity quite significantly influenced the results for PVA, Figure 10 shows that, in case of PVP, both trimers provided nearly identical results. This can be attributed to the fact that, unlike PVA, PVP does not exhibit intricate HB behavior. Furthermore, we tested how the consideration of a pentamer instead of a trimer influences the CS_dsp_^FV^ results. Interestingly, the pentamer again produced slightly higher AAD than both trimers. However, unlike PVA, the use of the pentamer decreased the already underestimated compatibility compared to both trimers.

We also examined the “molecular” approach, in which the entire polymer macromolecule is considered in the σ-profile calculation, thus completely bypassing the replication method. Naturally, PVPK12 was chosen as a test polymer, because it is the smallest molecule among the considered polymers (2500 g mol^–1^). This made its single-point DFT/CPCM calculations of σ feasible (specifically, it took 20 CPU hours on an AMD EPYC 7543 2.80 GHz machine). To obtain its geometries, we selected three representative PVPK12 molecules with different conformations that resulted from a recent MD simulation study:^19^*st*-mol1 (stretched), *st*-mol2 (U-shaped), and *st*-mol3 (S-shaped) (more details are given in Figure S14). Interestingly, the σ-profiles and solubility predictions based on these three conformations were quite similar, as shown in Figures 10 and S14. Specifically, their AAD values ranged from 8% to 10%, which means they were between those of the considered trimers and pentamer and comparable with them. The results based on the molecular approach were closer to those of the pentamer than to those of the trimers, which appears logical. However, for both PVP and PVA, it was one of the trimers, i.e., the shortest oligomer considered, that provided the best results in terms of AAD. Although this can be considered surprising, it is not abnormal, as earlier observations^62^ indicate that the increasing number of units in an oligomer within the replication approach does not always lead to a monotonous response or improvement of thermodynamic properties. The applied modeling approach includes many aspects and simplifications and, thus, the oligomer length in the polymer σ-profile calculation is not the only factor that determines its performance.

In general, the determination of a structure representative for a macromolecule can be a challenging task.^19^ In this context and considering the observations made for PVA and PVP, the replication approach on the basis of trimers for homopolymers, in combination with a reasonable selection of the oligomer tacticity (at least in case of intra-HB polymers), appears to be a meaningful strategy.

### 3.4 Enhancing Research Transparency with COSMOPharm

For the purpose of enabling and encouraging interested readers to reproduce our results or even produce their own predictions according to their preferences, an open-source Python-based tool COSMOPharm was developed. The package was crafted to provide a reproducible workflow for the application of COSMO-SAC models in predicting API–polymer compatibility. While COSMOPharm serves as a robust example of implementing COSMO-SAC for pharmaceutical ASD, it is primarily designed for interested readers to replicate some of the manuscript’s findings and to engage with the underlying models in a practical, hands-on manner. The tool incorporates several key features to enhance its functionality:

(1) **Extension of Original COSMO-SAC Functionality**: COSMOPharm extends the capabilities of the original open-source COSMO-SAC package,^50^ including the incorporation of the FV term to improve accuracy for polymer systems.
(2) **Solubility Calculation (Auto-SLE)**: With COSMOPharm, solubility calculations are streamlined through automatic SLE calculation, facilitating efficient prediction of API–polymer solubility. This method can also be used for API solubility in low-*M* solvents, extending its utility beyond polymer systems.
(3) **Miscibility Prediction (Auto-LLE)**: COSMOPharm includes an automated LLE calculation, enabling quick assessment of API–polymer AAPS, including both binodal and spinodal curves.

The COSMOPharm package, along with its source code, documentation, and usage examples, is openly available for academic and research purposes at https://github.com/ivanantolo/cosmopharm.

In addition to the core functionalities of COSMOPharm, the package also provides several resources to aid in the validation and reproduction of the results discussed in this manuscript. These additional materials include:

- The to_sigma_poly.py script, accompanied by validation data, to facilitate the generation of the σ-profiles of polymers from .cosmo files of shorter oligomers.
- .sigma files for all molecular species considered in this work, which are essential for testing the COSMO-SAC predictions and for recreating the present results.
- Exemplary Gaussian 16 input files for geometry optimization and CPCM single-point calculation of σ.
- A DirectImport C++ extension code is also included, offering a more convenient method for importing σ-profiles into the computational workflow of the COSMO-SAC package.
- Spreadsheets with all numerical results of this work.

These resources are intended to provide a comprehensive toolkit for researchers and practitioners interested in exploring the application of COSMO-SAC models in the pharmaceutical field, enhancing the reproducibility and accessibility of the research findings.

For clarity, the current limitations of COSMOPharm with respect to ASD, as of July 2024, are the following:

- Only binary API–polymer systems are considered. The effects of a third component of ASD, such as water (moisture) or residual solvent, are not included yet.
- Regarding polymers, only monodisperse systems with molecules of the same chain length are considered.
- Only linear polymers are considered. Modified polymers, such as branched, grafted, and cross-linked polymers, are not accounted for yet; neither is the swelling effect of polymers.
- In case of copolymers, at most two distinct monomer units can be considered.

However, many of these limitations may be addressed in future efforts. It is also important to note that COSMOPharm, as a tool for predicting *equilibrium* properties using the COSMO-SAC *thermodynamic* model, cannot simulate detailed process-related factors^65,105,111,116,117^ or account for spatial and temporal (i.e., kinetic) effects.

## 4 Conclusion

The capabilities and limitations of the COSMO-SAC model, with a particular focus on its COSMO-SAC-dsp variant in conjunction with the FV term, were comprehensively investigated for predicting the equilibrium solubility and miscibility of API in various polymers, a key aspect in the formulation of ASD. Through a detailed examination, significant insights into quantitative and qualitative aspects of model performance, the impact of modeling choices, and the importance of different model components were gleaned.

Quantitatively, it was found that the COSMO-SAC model offers a reasonable estimation of API solubility in polymers, with the overall AAD(*w*_API_) from experimental data being 13%. However, the performance varies significantly across different API–polymer systems. The model generally overestimates API solubility, especially in systems with low solubility values, although this tendency was not uniformly observed across all API or polymers.

Qualitatively, the model demonstrated strong capabilities in predicting AAPS and rank-ordering polymer candidates according to their thermodynamic compatibility with API. This underlines the utility of COSMO-SAC in the early stages of formulation development for (pre)screening suitable polymers and identifying polymers with the best and worst compatibility with API, thus guiding formulation strategies effectively.

A sensitivity analysis with respect to the σ-profiles of polymers and model configuration was performed. The replication approach using trimers proved to be a meaningful strategy for σ-profiles of homopolymers. The inclusion of the FV term was observed to slightly improve the numerical accuracy of solubility predictions compared to CS_dsp_^SG^. However, this improvement is not as significant as might be expected, and the similarity of the FV ratios of API and polymers was identified as the main reason for this observation. Moreover, the dispersion contribution was found to be even more negligible than FV. This suggest that the standard COSMO-SAC configuration with the SG term (whether with the dispersion term or without it) could be a viable alternative for ASD. To contextualize these findings and the observed predictive performance, the total AAD of 14% obtained from the fully QM-aided CS_dsp_^SG^ model was about 10% lower than that of other available models for ASD phase behavior.^41^ It requires no experimental data for parametrization because it only relies on first-principles calculations, reflecting its practical efficacy.

In this context, COSMOPharm was designed as a practical and accessible tool for researchers to replicate the findings of this study and explore API–polymer compatibility. It aims to incorporate features highlighted by Turpin et al.,^21^ such as QM-reliance and scriptability. Utilizing the open-source COSMO-SAC package,^50^COSMOPharm streamlines the application of these insights, facilitating the advancement of pharmaceutical formulation technology.

This research highlights the dual nature of the impact of COSMO-SAC: its significant qualitative insights for polymer compatibility screening and the need for mindful consideration of its quantitative limitations. The model’s role in simplifying the early stages of formulation development, particularly in aiding the selection of polymer carriers, can be substantial. Enhancing the model with more detailed polymer characteristics and process-related factors stands out as a crucial step toward improving its accuracy and broadening its application in formulating advanced drug delivery systems.

## Data Availability

The current version of the COSMOPharm package (Section 3.4) can be found at https://github.com/ivanantolo/cosmopharm. The archival version of COSMOPharm used in this paper is furthermore stored at 10.5281/zenodo.10792203.

## Supporting Information Available

Chemical identifiers of the considered API and polymers; Illustrative phase diagrams for ASD; Parameters of the free volume term; Determined σ-profiles of API and polymers; Visualization of statistical deviations and their correlation with molecular descriptors; Results of AAPS analysis and technical details regarding polymer ranking; Supporting results and discussions on sensitivity analysis (PDF)
