# Supplementary material for: COSMOPharm: Drug–Polymer Compatibility of Pharmaceutical Amorphous Solid Dispersions from COSMO-SAC
Source: Mol Pharm. 2024 Jul 30;21(9):4395–415. doi: 10.1021/acs.molpharmaceut.4c00342 (PMC11372840; doi:10.1021/acs.molpharmaceut.4c00342)
Supplement: Supplementary file 1 — mp4c00342_si_001.pdf [file mp4c00342_si_001.pdf]

Supporting Information

COSMOPharm: Drug–Polymer Compatibility of Pharmaceutical Amorphous Solid Dispersions from COSMO-SAC

Ivan Antolović,[†] Jadran Vrabec,[†] and Martin Klajmon[*,‡]

†*Thermodynamics, Technische Universität Berlin, Ernst-Reuter-Platz 1, 10587 Berlin, Germany*

‡*Department of Physical Chemistry, University of Chemistry and Technology, Prague, Technická 5, 166 28 Prague 6, Czechia*

E-mail: martin.klajmon@vscht.cz

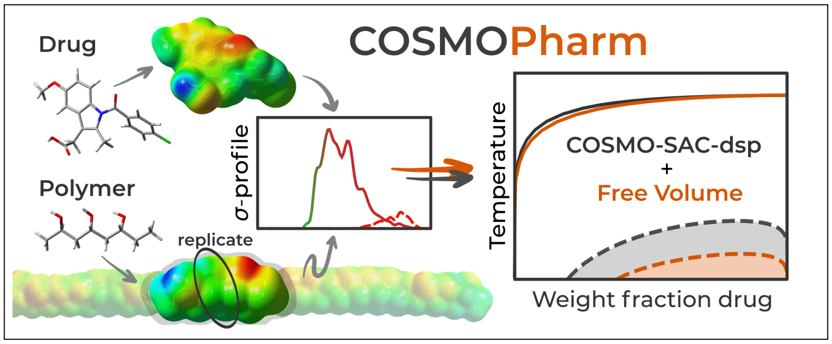

S1 Chemical Identifiers of API and Polymers

Table S1: Considered API and their chemical identifiers

API	Abbr.	$M/(\text{g mol}^{-1})$	CAS RN
Griseofulvin	GSF	352.77	126-07-8
Ibuprofen	IBP	206.28	15687-27-1
Indomethacin	IMC	357.79	53-86-1
Nifedipin	NIF	346.34	21829-25-4
Naproxen	NPX	230.26	22204-53-1
Paracetamol	PCM	151.16	103-90-2
Simvastatin	SIM	418.60	79902-63-9

Table S2: Considered polymers and their chemical identifiers

Polymer[a]	Abbr.	$M/(\text{g mol}^{-1})$	Monomer(s)	CAS RN	w_{mono}[b]	N_{units}[b]
EUDRAGIT® L100-55	EUD	212000	methacrylic acid (PMAA)	79-41-4	0.462	1140
			ethyl acrylate (PEA)	140-88-5	0.538	1140
PDLLA	PDL	16400	lactic acid (PLA)	50-21-5	1	228
PLGA 50:50[c]	PLGA50	9877	lactic acid (PLA)	50-21-5	0.554	76
			glycolic acid (PGA)	79-14-1	0.446	76
PLGA 75:25[c]	PLGA75	12900	lactic acid (PLA)	50-21-5	0.788	141
			glycolic acid (PGA)	79-14-1	0.212	47
PVA (Parteck® MXP)	PVA	32000	vinyl alcohol (PVA)	9002-89-5	1	726
PVP K12[c]	PVPK12	2500	*N*-vinyl-2-pyrrolidone (PVP)	88-12-0	1	22
PVP K25[c]	PVPK25	25700	*N*-vinyl-2-pyrrolidone (PVP)	88-12-0	1	231
PVP K30[c]	PVPK30	49000	*N*-vinyl-2-pyrrolidone (PVP)	88-12-0	1	441
Kollidon® VA64	PVPVAc64	65000	*N*-vinyl-2-pyrrolidone (PVP)	88-12-0	0.4	351
			vinyl acetate (PVAc)	108-05-4	0.6	302

[a]Commercial/established names. Acronyms: PDLLA = poly(D,L-lactic acid); PLGA = poly(D,L-lactic-co-glycolic acid); PVA = poly(vinyl alcohol); PVP = poly(vinyl pyrrolidone).
[b]Weight fraction of a monomer unit in the polymer molecule: $N_{\text{units}} = (w_{\text{mono}} M)/M_{\text{mono}}$ (for homopolymers, $w_{\text{mono}} = 1$).
[c]Note that the two PLGA polymers differ in both the ratio of monomer units and chain length (*i.e.*, M), while the three PVP polymers only differ in M.

S2 Phase Diagrams for ASD

The phase diagrams, which comprise both the calculated SLE and LLE curves, are the basis for the prediction of API–polymer compatibility. Illustrative examples of API–polymer phase diagrams are shown in Figure S1 for three cases with varying degree of compatibility between API (acting as a solute) and polymer (acting as a solvent). It depicts the equilibrium solubility line of a crystalline API in an amorphous/molten polymer under SLE. Compositions above and to the left of the solubility line denote thermodynamically stable homogeneous ASD undersaturated with respect to the crystalline API. Below the solubility line, the systems are metastable, supersaturated with respect to the API, and the API tends to (re)crystallize to achieve the equilibrium API solubility value. If kinetic hindrance is overcome, this will reduce the amount of API in a homogeneous ASD.

Systems with a high API–polymer compatibility are often completely miscible (Figure S1a), while those with a low compatibility exhibit not only reduced API solubility, but sometimes also a limited API–polymer miscibility, leading to AAPS. This is manifested by the presence of an LLE binodal curve (Figures S1b and c) which envelops the demixing region and indicates the compositions of the two coexisting amorphous phases (one with

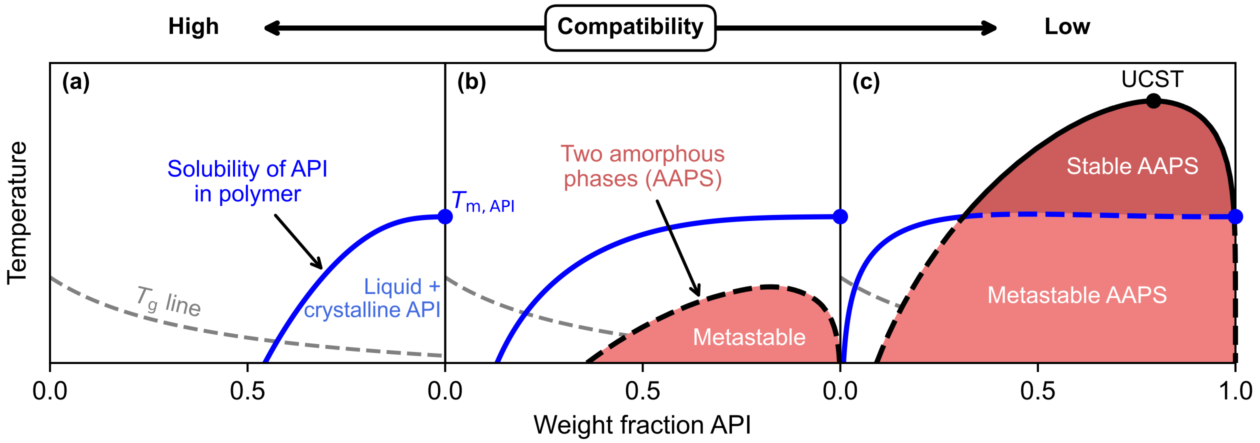

Figure S1: Schematic phase diagrams of API–polymer systems with three different levels of compatibility. Note that solubility (blue line) and miscibility (black line) decline with decreasing compatibility, *i.e.*, from (a) to (c). UCST denotes the upper critical solution temperature, the maximum temperature where AAPS exists.

a higher and one with a lower API weight fraction). In general, the occurrence of AAPS negatively impacts the thermodynamic stability of ASD. Note that the AAPS envelope (or, at least, a part of it) is often found below the solubility line. The two coexisting amorphous phases are then metastable with respect to API crystallization, as indicated in Figure S1. This means that the phases have a limited lifetime and tend to homogenize into a single stable amorphous phase (with w_{API} corresponding to the equilibrium API solubility) that is in equilibrium with the crystalline API. In contrast, AAPS above the solubility line leads to two stable amorphous phases, as shown in Figure S1c.

However, both the crystallization and phase separation can be kinetically hindered and delayed due to the large viscosity that ASD typically exhibit. Further kinetic hindrance occurs when the temperature falls to the vicinity of, and below, the T_{g} line, where molecules are almost immobilized (it should be pointed out that T_{g} cannot be calculated with COSMO-SAC and is not considered in this work). Therefore, even systems in a thermodynamically metastable state can remain "kinetically" stabilized and homogeneous over long periods of time.[1,2]

A more comprehensive description and general discussion about thermodynamic equilibrium phase diagrams in binary polymer systems can be found in Prausnitz *et al.*,[3] Burghardt,[4] or Byun and Lee.[5]

S3 Parameters of the Free Volume Term

Table S3: Molar liquid volume (v)[a], hard core volume (v^{HC})[b], free volume (v^{F})[c], and v^{F}/v of API and polymers considered in this study (in $\mathrm{cm}^3\ \mathrm{mol}^{-1}$)

Compound	v	v^{HC}	v^{F}	$100 \cdot (v^{\mathrm{F}}/v)$	Source of v
			API		
GSF	281.77	184.73	97.04	34.4	(ref[6])[d]
IBP	204.46	130.79	73.68	36.0	ref[7]
IMC	269.20	183.70	85.50	31.8	(ref[7])[e]
NPX	192.79	129.32	63.47	32.9	(ref[7])[e]
NIF	266.42	193.08	73.34	27.5	ref[8]
PCM	121.35	85.33	36.01	29.7	(ref[7])[e]
SIM	397.15	263.34	133.82	33.7	(ref[6])[d]
			Polymers		
EUD	198131	127409	70722	35.7	ref[9]
PDL	13120	9202	3918	29.9	ref[10]
PLGA50	7902	5352	2550	32.3	ref[10]
PLGA75	10320	7103	3217	31.2	ref[10]
PVA	24480	18993	5487	22.4	ref[11]
PVPK12	2101	1464	637	30.3	ref[12]
PVPK25	21780	15129	6651	30.5	ref[13]
PVPK30	43750	28859	14891	34.0	ref[13]
PVPVAc64	60185	38275	21910	36.4	ref[14]

[a] The values of v correspond to a temperature of 298 K.
[b] The values of v^{HC} correspond to van der Waals volumes based on Bondi radii[15] and were determined using the fast calculation method proposed by Zhao *et al.*[16]
[c] $v^{\mathrm{F}} = v - v^{\mathrm{HC}}$
[d] Estimated using a quantitative structure–property relationship (QSPR) approach implemented in the Amsterdam Modeling Suite, version 2022.101.[6]
[e] Experimental data extrapolated to 298 K.

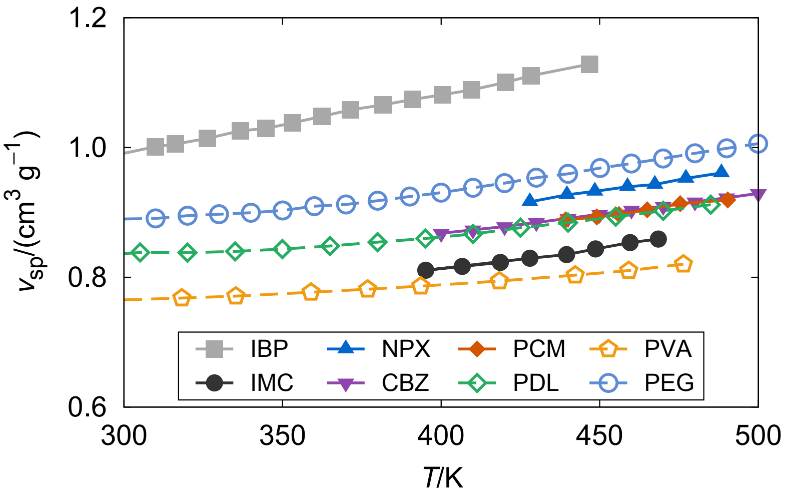

Figure S2: Specific volume as a function of temperature for selected liquid/amorphous API and polymers at 0.1 MPa. Data sources: IBP, IMC, NPX, and PCM – experimental data[7]; PVA – experimental data[11]; carbamazepine (CBZ; an API), PDL and poly(ethylene glycol) (PEG) – molecular dynamics simulations[17,18]. The lines are a guide the eye.

S4 σ-Profiles of API and Polymers

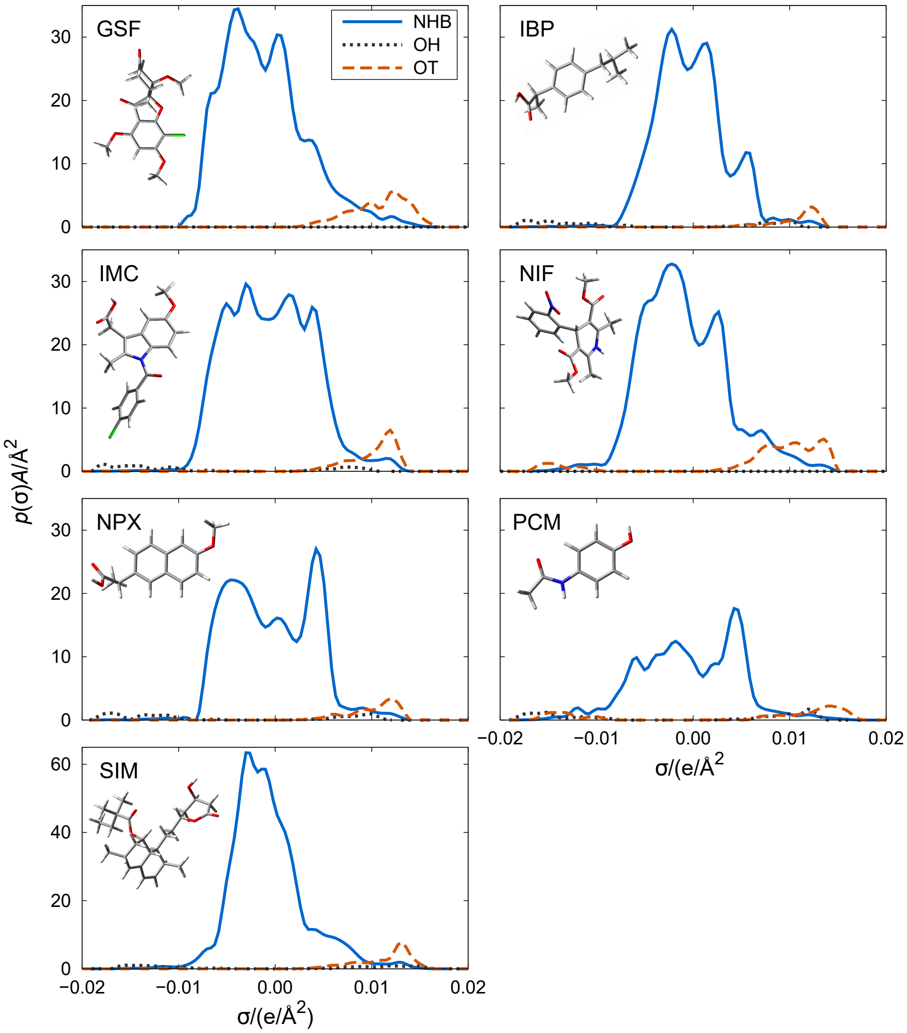

Figure S3: Calculated σ-profiles for the API considered in this work. Solid lines, non-hydrogen-bonding part of the σ-profile (NHB); dotted lines, hydrogen-bonding part due to hydroxyl groups (OH); dashed lines, hydrogen-bonding part due to other groups (OT). Note the different vertical axis range in case of SIM. The graphs also show the optimized molecular geometries of the API.

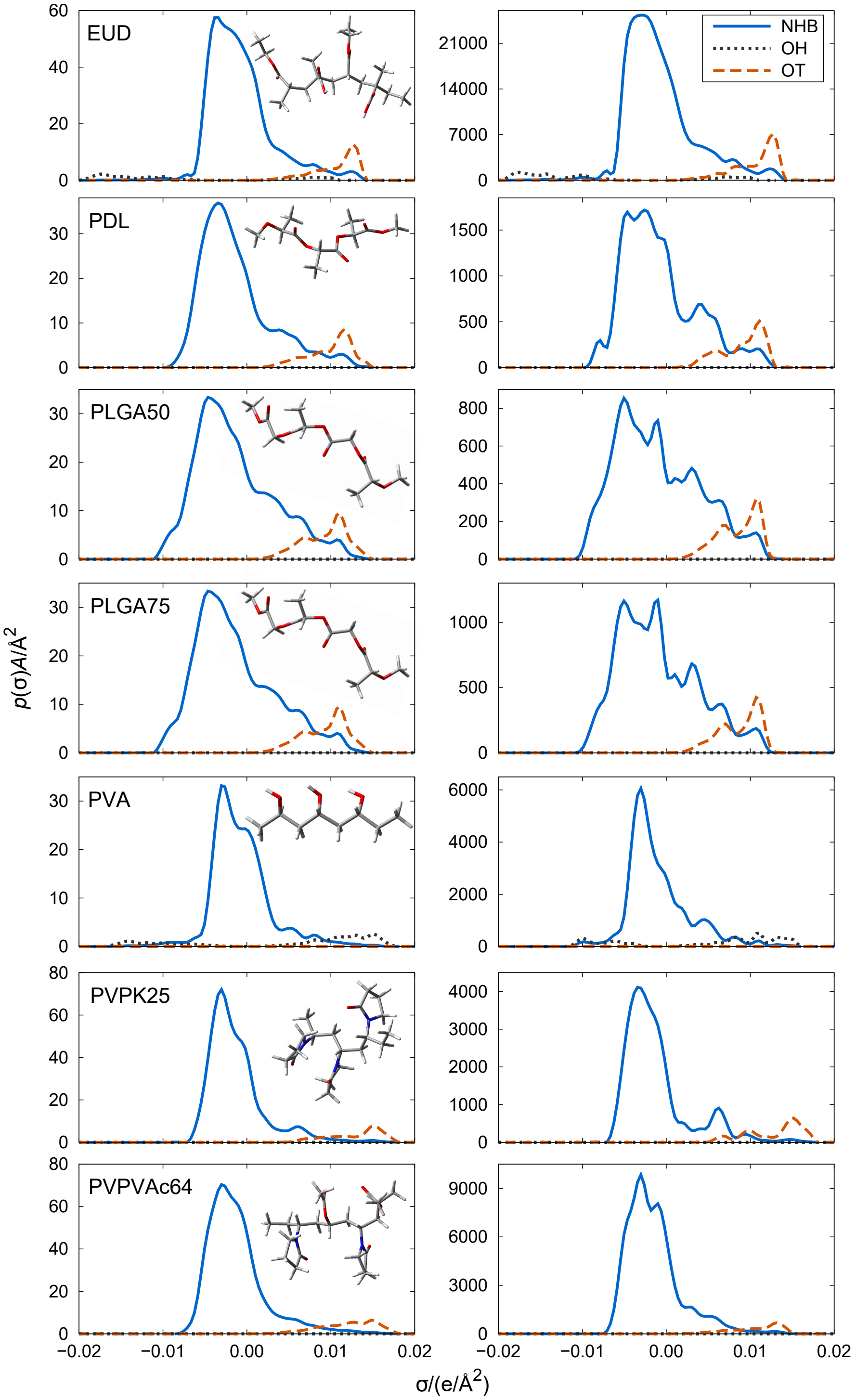

Figure S4: Calculated σ-profiles for the oligomers (left column) and the respective polymers (right column) considered in this work. In case of the PVP polymers, only PVPK25 is shown as a representative. For the legend, see the caption of Figure S3. The graphs in the left column also show the optimized molecular geometries of the oligomers.

S5 Analysis of Quantitative Performance

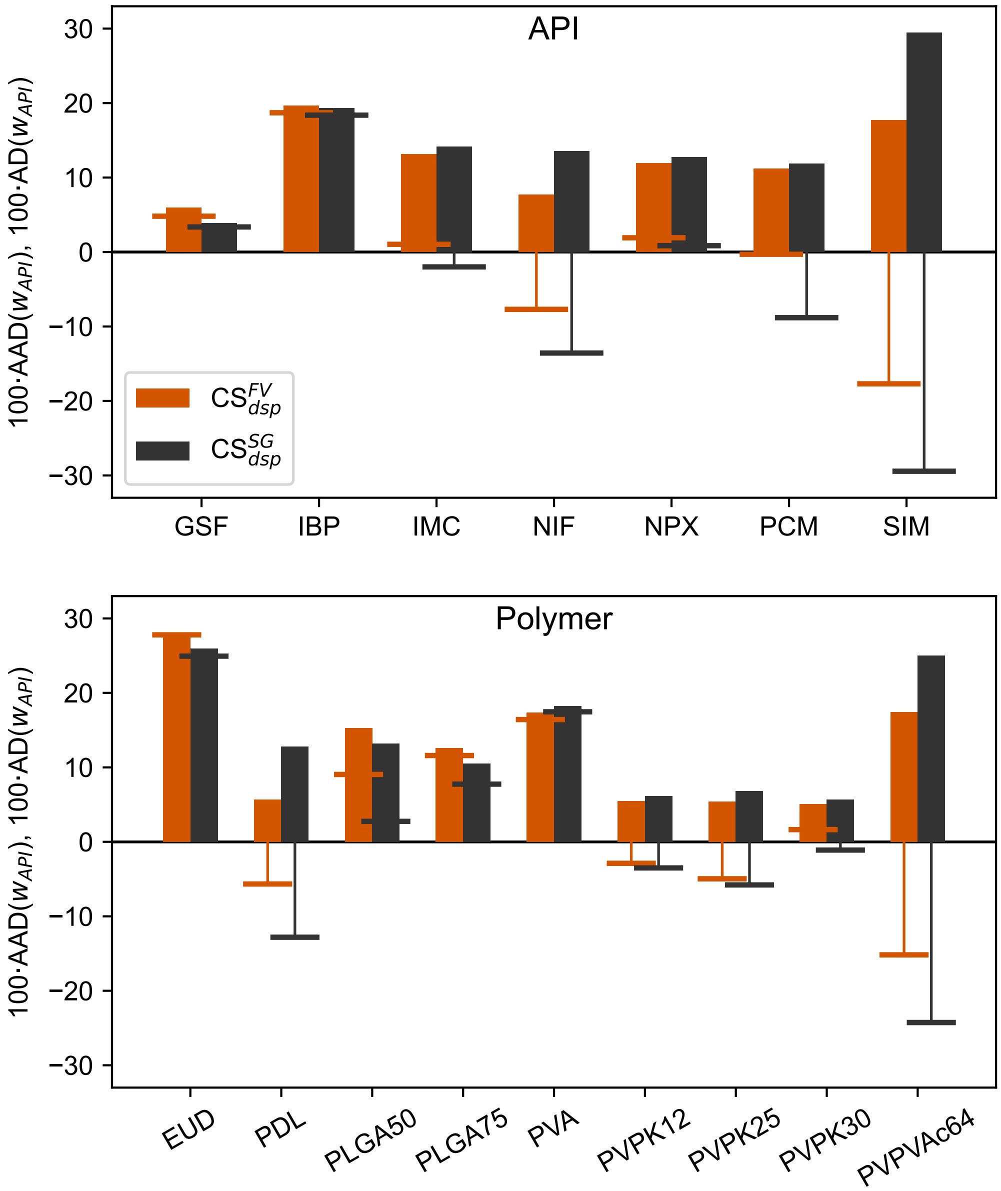

Figure S5: Overview of AAD(w_{API}) and AD(w_{API}) values obtained in this work for the API solubility predicted by $\mathrm{CS}^{\mathrm{FV}}_{\mathrm{dsp}}$ and $\mathrm{CS}^{\mathrm{SG}}_{\mathrm{dsp}}$ for (top) each of the considered API over all solvents and (bottom) each of the considered polymers over all API. The bars represent AAD (always positive values), while the horizontal lines show the corresponding AD.

S5.1 Correlations of the Prediction Error with Selected Substance Descriptors

The molar mass serves as a rough indicator of the molecular size of both polymers and API (and also structural complexity, especially in the case of API[19]) and, as such, it is typically one of the most significant descriptors regarding API–polymer systems.[20] First, a moderate correlation ($R^2 = 0.59$; R^2 is the coefficient of determination) was found between AAD(w_{API}) calculated over individual polymers and their molar mass (Figure S6a). However, if EUD with its outstanding M value of 212 000 g mol^{-1} is excluded from the analyzed set, the R^2 value drops drastically to only 0.09, which indicates that there is no significant correlation between AAD and M for the other polymers. Similarly, Figure S6b shows no correlation between AAD and M_{API}, but it can at least be seen that it is not the API with the highest M values that are associated with the largest errors. The correlation of AAD with the API melting point temperature shown in Figure S6c demonstrates a significantly stronger relationship and indicates the overall error to decrease with $T_{\mathrm{m,API}}$. Thus, the last two correlations regarding API suggest that an increasing API complexity does not lead to larger error.

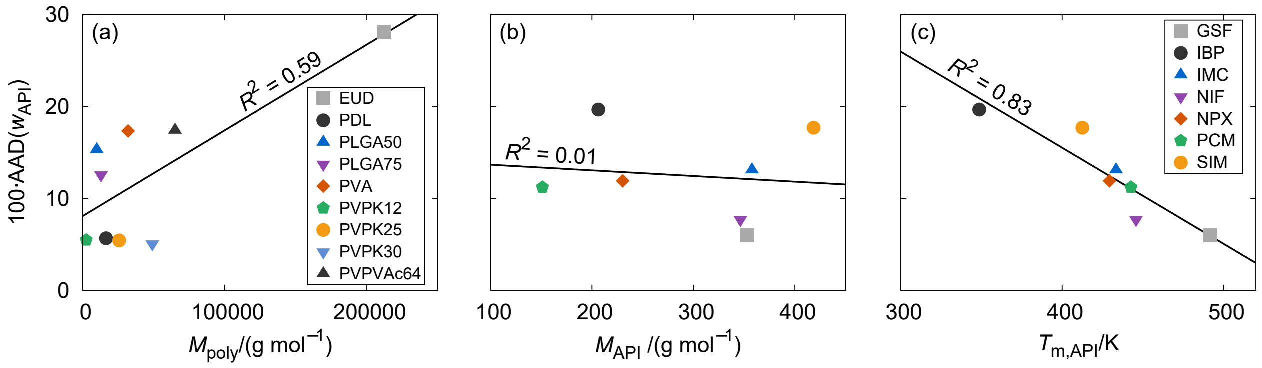

Figure S6: Correlation of AAD(w_{API}) derived from $\mathrm{CS}^{\mathrm{FV}}_{\mathrm{dsp}}$ with (a) polymer molar mass, (b) API molar mass, and (c) API melting point temperature. The black line depicts regressions.

S6 AAPS Analysis

Table S4: Computational analysis[a] of AAPS predicted with CS^{FV}_{dsp}

System	Exp. evidence	CS^{FV}_{dsp}	LLE type predicted
GSF–PVPK12	× [21]	×	
GSF–PVPK25	× [21]	×	
GSF–PVPK30	× [21]	×	
GSF–PVPVAc64	× [21]	×	
IBP–EUD	× [9]	×	
IBP–PLGA50	✓ [2]	✓	UCST ≈ 300 K
IBP–PLGA75	✓ [2]	✓	UCST ≈ 260 K
IBP–PVA	n/a	×	
IBP–PVPK12	× [21]	×	
IMC–EUD	n/a	×	
IMC–PLGA50	× [2]	✓	UCST ≈ 190 K
IMC–PLGA75	× [2]	×	
IMC–PVA	× [22]	×	
IMC–PVPK12	× [23]	×	
IMC–PVPVAc64	× [14]	×	
NIF–PVPK12	× [21]	×	
NIF–PVPK25	× [21]	×	
NIF–PVPK30	× [21]	×	
NIF–PVPVAc64	× [21]	×	
NPX–EUD	n/a	×	
NPX–PLGA50	× [2]	✓	LCST < UCST ≈ 135 K[b]
NPX–PLGA75	✓ [2]	×	
NPX–PVA	✓ [22]	×	
NPX–PVPK12	× [21]	×	
NPX–PVPK25	× [21]	×	
NPX–PVPK30	× [21]	×	
NPX–PVPVAc64	× [21]	×	
PCM–EUD	n/a	×	
PCM–PLGA50	× [2]	✓	LCST < UCST ≈ 175 K[b]
PCM–PLGA75	× [2]	✓	LCST < UCST ≈ 155 K[b]
PCM–PVPK12	× [21]	×	
PCM–PVPK30	× [24]	×	
PCM–PVPVAc64	× [24]	×	
SIM–PDL	n/a	✓	UCST ≈ 330 K
SIM–PLGA50	n/a	✓	UCST ≈ 430 K

[a] Symbols and acronyms: × = AAPS not observed/predicted; ✓= AAPS observed/predicted; n/a = not available (AAPS analysis has not yet been performed).
[b] Low-temperature closed-loop LLE with both LCST and UCST.

S7 Polymer Ranking: Technical Details

As outlined in the main article, for each API, both the predicted and experiment-based polymer orders were primarily determined from API solubility values in polymers (at the lowest experimental temperature reported for a given API). For example, in case of NPX, the evaluation was performed at about 373 K, corresponding to the NPX–PVPVAc64 system. The lowest temperature was chosen because it is closest to the storage temperature (≈ 298 K), which is of interest for pharmaceutical applications. For systems where experimental temperature range did not include this temperature, the binary interaction parameters (k_{ij}) of the PC-SAFT equations of state[19,25] were adjusted using the experimental solubility data. Having these experiment-based PC-SAFT correlations with fitted k_{ij} allowed for the extrapolation of the experimental solubility data to lower temperatures and, particularly, to the lowest experimental T, where the polymer order was determined. Therefore, we denote it "experiment-based" order, to emphasize the fact that the experimental data were not used directly but underwent an extrapolation procedure. In principle, the same procedure to determine the experiment-based polymer order using PC-SAFT extrapolations has already been used in other recent studies.[2,19,26,27] Moreover, when either experiment or prediction attributed AAPS behavior to a system, the associated polymer automatically incurred a penalty, which resulted in its position being shifted one place down in the corresponding compatibility order.

S8 Sensitivity Analysis: Combinatorial Terms

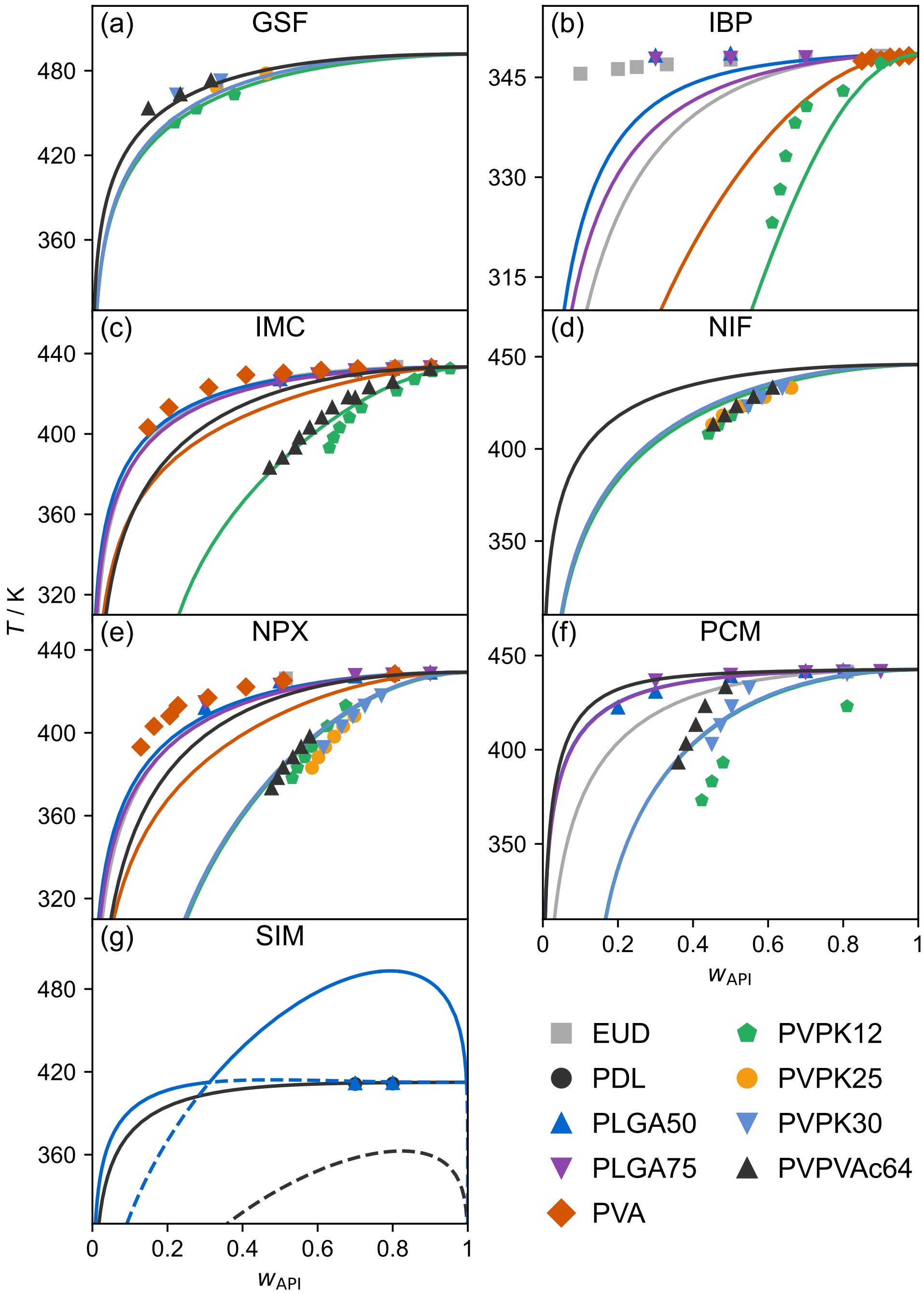

Figure S7: Solubility curves (solid lines) and AAPS curves (dashed lines) predicted by CS^{SG}_{dsp} compared to experimental solubility data (symbols).

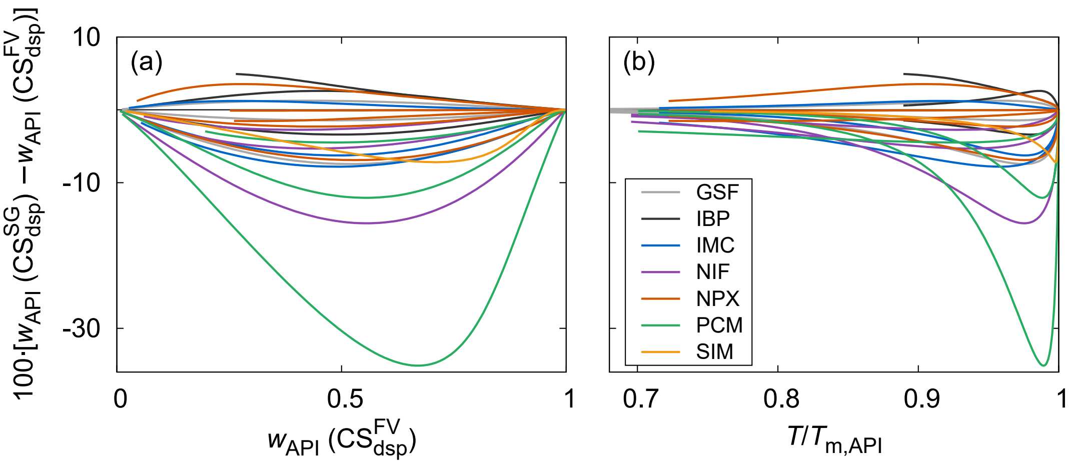

Figure S8: Sensitivity analysis regarding the combinatorial contributions in terms of the calculated API solubility values: difference between CS_{dsp}^{SG} and CS_{dsp}^{FV} against (a) w_{API} (CS_{dsp}^{FV}) and (b) $T/T_{m,API}$. For clarity, only 3 to 5 curves (*i.e.*, systems) per API were plotted. Since the SG and FV terms are temperature-independent, figure (b) only serves as an alternative projection of figure (a), using the temperatures for which the solubilities were calculated.

S8.1 Difference between $\mathbf{CS_{dsp}^{FV}}$ and $\mathbf{CS_{dsp}^{SG}}$ at Low Temperatures

Figure S9 shows contributions of the individual terms to the thermodynamic activity of the API ($\ln a_{API} = \ln x_{API} + \ln \gamma_{API}$) for the NIF–PVPK30 systems which exhibits a representative "parabolic" behavior regarding the difference between w_{API} from CS_{dsp}^{FV} and CS_{dsp}^{SG}. Figure S9a depicts the course of the individual terms at two temperatures: 310 K, which corresponds to low equilibrium w_{API} values and very small differences between CS_{dsp}^{FV} and CS_{dsp}^{SG}, and 400 K, where the difference between CS_{dsp}^{FV} and CS_{dsp}^{SG} was among the largest (closer to the middle of the concentration interval). Solving the API solubility equation at a given temperature is, in principle, searching for a x_{API} (or w_{API}) value where $\ln a_{API} = -\Delta_{fus} g_{API}/(RT)$ (*cf.* eq 1 in the main article). This is illustrated in Figure S9a by the intersections of the horizontal lines denoting $-\Delta_{fus} g_{API}/(RT)$ at each temperature and the (total) activity curves. It can be seen that the intersection points, where the activity attains the required value, are more distant from each other in terms of w_{API} at 400 K than at 310 K, where the distance between the equilibrium w_{API} values from CS_{dsp}^{FV} and CS_{dsp}^{SG} is small. This corresponds to the

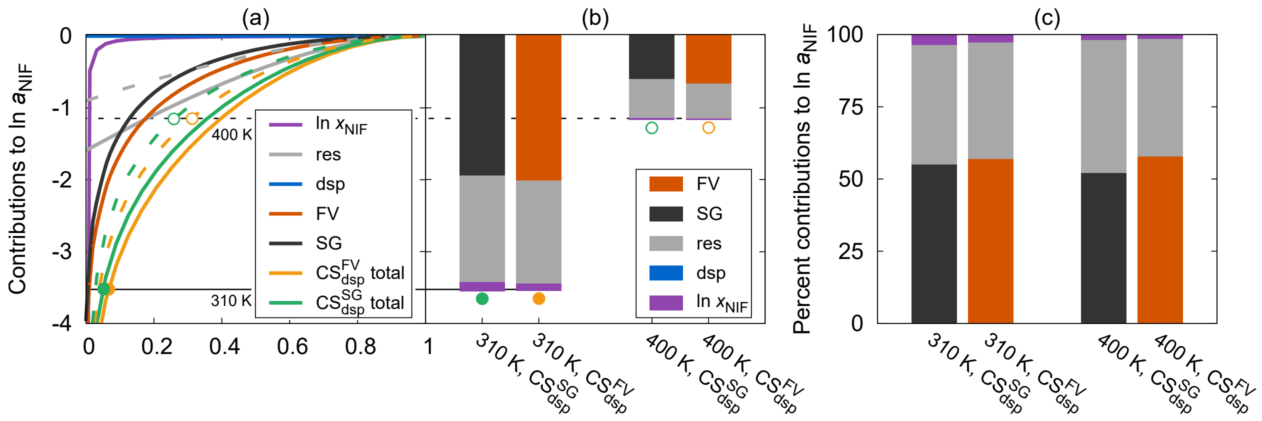

Figure S9: Individual contributions to the thermodynamic activity of API for the NIF–PVPK30 systems at temperatures of 310 K (solid lines) and 400 K (dashed lines): (a) contributions as a function of w_{API}, (b) contributions at equilibrium solubility (saturation), and (c) percent contributions at saturation.

observed differences in w_{API} values predicted by $\mathrm{CS}_{\mathrm{dsp}}^{\mathrm{FV}}$ and $\mathrm{CS}_{\mathrm{dsp}}^{\mathrm{SG}}$ at both temperatures and the observed parabolic behavior depicted in Figure S8. Furthermore, Figure S9b details the contributions of the individual terms to the total NIF activity value at the saturation points, while Figure S9c presents the same information in relative terms. At both temperatures, both combinatorial terms contribute about 50 % to 60 % to the total activity value; the rest is mainly due to the residual term. However, Figure S9c reveals that, although the relative contributions of the FV and SG terms at 310 K are almost comparable, the contribution of FV at 400 K is obviously higher than that of SG. This observation indicates that $\mathrm{CS}_{\mathrm{dsp}}^{\mathrm{FV}}$ attributed an appreciably more favorable behavior to this system with respect to the thermodynamic compatibility (*i.e.*, more negative entropic deviations from ideality), thus making the activity to achieve its equilibrium value at a higher NIF concentration.

S8.2 Difference between Free Volume Ratios of API and Polymers

Following the principles outlined in Section 3.3.1 of the main article, $\mathrm{CS}_{\mathrm{dsp}}^{\mathrm{FV}}$ should tend to provide a higher API solubility than $\mathrm{CS}_{\mathrm{dsp}}^{\mathrm{SG}}$ for systems where $\left(v^{\mathrm{F}}/v\right)_{\mathrm{API}} < \left(v^{\mathrm{F}}/v\right)_{\mathrm{poly}}$, due to the gain in FV upon mixing compared to the pure API (the prevailing phenomenon observed in this work), and *vice versa*. The systems considered in this study roughly follow

this behavior, as illustrated in Figure S10, where the average difference between w_{API} from $\mathrm{CS}_{\mathrm{dsp}}^{\mathrm{FV}}$ and $\mathrm{CS}_{\mathrm{dsp}}^{\mathrm{SG}}$ is plotted against the difference between v^{F}/v of the API and polymers. (The reason why not all systems strictly follow this principle can be attributed to differences between FH and SG and between v^{HC} values used in FV and SG.) For instance, systems contradicting the prevailing observation of $\mathrm{CS}_{\mathrm{dsp}}^{\mathrm{SG}}$ producing lower w_{API} than $\mathrm{CS}_{\mathrm{dsp}}^{\mathrm{FV}}$, *i.e.*, GSF- and PVA-based systems, have $\left(v^{\mathrm{F}}/v\right)_{\mathrm{API}} > \left(v^{\mathrm{F}}/v\right)_{\mathrm{poly}}$ in most cases. The case of PVA is particularly obvious in this regard because it possess the lowest v^{F}/v value among all substances considered in this work (22.4 %). This results in its mixing with any API being associated with a loss in FV from the API perspective and, therefore, $\mathrm{CS}_{\mathrm{dsp}}^{\mathrm{SG}}$ produces higher w_{API} than $\mathrm{CS}_{\mathrm{dsp}}^{\mathrm{FV}}$.

Similarly, the largest difference between $\mathrm{CS}_{\mathrm{dsp}}^{\mathrm{FV}}$ and $\mathrm{CS}_{\mathrm{dsp}}^{\mathrm{SG}}$ encountered for the PCM–PVPVAc64 system can be rationalized by the fact that the v^{F}/v value of PCM (29.7 %; see Table S3) is one of the lowest among the API, while that of PVPVAc64 (36.4 %) is the highest with respect to the polymers, making their difference one of the largest. Since $\mathrm{CS}_{\mathrm{dsp}}^{\mathrm{SG}}$ substantially underestimates the API solubility in this case, switching to $\mathrm{CS}_{\mathrm{dsp}}^{\mathrm{FV}}$, together with the fact that $\left(v^{\mathrm{F}}/v\right)_{\mathrm{PCM}} < \left(v^{\mathrm{F}}/v\right)_{\mathrm{PVPVAc64}}$, leads to a significant increase of the calculated solubility and improved performance in terms of AAD.

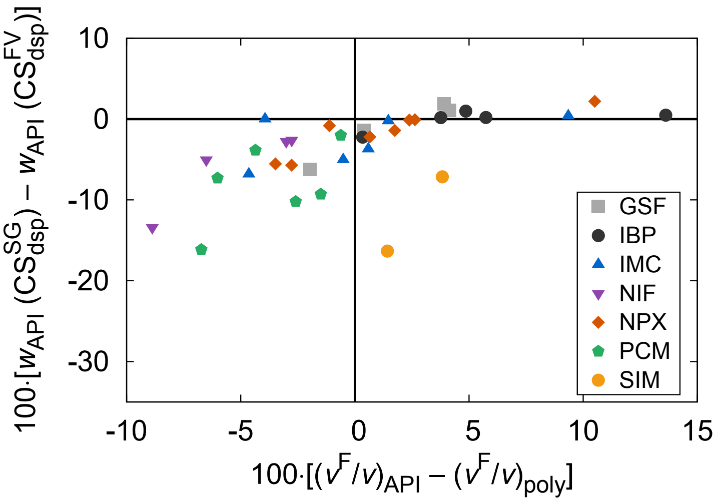

Figure S10: Average difference between $\mathrm{CS}_{\mathrm{dsp}}^{\mathrm{SG}}$ and $\mathrm{CS}_{\mathrm{dsp}}^{\mathrm{FV}}$ against difference between $\left(v^{\mathrm{F}}/v\right)_{\mathrm{API}}$ and $\left(v^{\mathrm{F}}/v\right)_{\mathrm{poly}}$ for all systems studied in this work.

S9 Sensitivity Analysis: Dispersion Term

Trying to rationalize the negligible importance of the dispersion term to the results for ASD, we plotted the difference between w_{API} values calculated from $\mathrm{CS}^{\mathrm{FV}}$ and $\mathrm{CS}^{\mathrm{FV}}_{\mathrm{dsp}}$ against $w_{\mathrm{API}}(\mathrm{CS}^{\mathrm{FV}}_{\mathrm{dsp}})$ in Figure S11b. Unlike the combinatorial contributions $\mathrm{CS}^{\mathrm{FV}}_{\mathrm{dsp}}$ and $\mathrm{CS}^{\mathrm{SG}}_{\mathrm{dsp}}$, the difference between $\mathrm{CS}^{\mathrm{FV}}$ and $\mathrm{CS}^{\mathrm{FV}}_{\mathrm{dsp}}$ shows no parabolic behavior, but gradually rises with decreasing w_{API} (thus, increases with falling temperature). In the asymmetric API–polymer systems ($M_{\mathrm{API}} \ll M_{\mathrm{poly}}$), it is important to realize that even relatively low w_{API} values correspond, in fact, to large x_{API} values. Considering GSF–PVPK30 ($M_{\mathrm{PVPK30}} = 49000$ g mol^{-1}) as an example, a GSF weight fraction value of 0.5 corresponds to $x_{\mathrm{GSF}} = 0.993$, the lowest experimental w_{API} value of 0.224 corresponds to $x_{\mathrm{GSF}} = 0.976$, and even $w_{\mathrm{GSF}} = 0.1$ still means $x_{\mathrm{GSF}} = 0.939$. In other words, the majority of the data points at T_{exp} is, in terms of x_{API}, shifted toward the infinite dilution state of the polymer in the API, *i.e.*, $x_{\mathrm{API}} \to 1$ and $x_{\mathrm{poly}} \to 0$ (and this shift is more significant the larger M_{poly} is). Therefore, $\ln \gamma_i^{\mathrm{dsp}}$ can roughly be approximated by their limiting values over a relatively broad range of w_{API} (for clarity, none of the following approximation was used in our calculations)[28]

$$\begin{aligned} \lim_{x_{\mathrm{API}}\to 1} \ln \gamma_{\mathrm{API}}^{\mathrm{dsp}} &= 0 \\ \lim_{x_{\mathrm{API}}\to 1} \ln \gamma_{\mathrm{poly}}^{\mathrm{dsp}} &= A \end{aligned} \tag{S1}$$

The zero value of the limiting $\ln \gamma_{\mathrm{API}}^{\mathrm{dsp}}$ can explain the negligible difference between $\mathrm{CS}^{\mathrm{FV}}$ and $\mathrm{CS}^{\mathrm{FV}}_{\mathrm{dsp}}$ at higher $w_{\mathrm{API}}(\mathrm{CS}^{\mathrm{FV}}_{\mathrm{dsp}})$ values. The increase of the difference with rising w_{API} then manifests the vanishing relevance of the limiting assumption (eq S1) and increasing importance of the dispersion contribution. However, its numerical effect on the overall results is very small even at low w_{API}.

What also arises from the combined effect of the difference between w_{API} and x_{API} and the simple Margules-like form of $\ln \gamma_i^{\mathrm{dsp}}$ is that the dispersion term has a different importance for systems with the different PVP polymers. For example, the average difference between

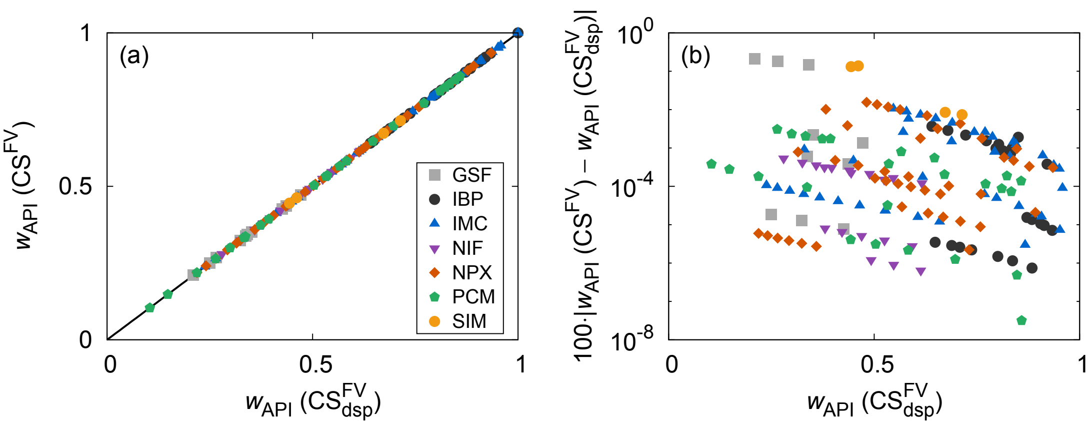

Figure S11: Sensitivity analysis regarding the dispersion contribution in terms of the calculated API solubility values: (a) CS^{FV} *vs.* CS^{FV}_{dsp} by means of a diagonal graph and (b) difference between CS^{FV} and CS^{FV}_{dsp} as a function of w_{API} (CS^{FV}_{dsp}). Note the logarithmic scale of the vertical axis used in the latter. The data points were calculated at T_{exp}.

w_{API} values calculated by CS^{FV} and CS^{FV}_{dsp} is 0.02 %, $(3 \cdot 10^{-4})$%, and $(7 \cdot 10^{-5})$% for all systems with PVPK12, PVPK25, and PVPK30, respectively. This would mean that, if we take two systems, *e.g.*, GSF–PVPK12 and GSF–PVPK30, each with equimass composition ($w_{API} = 0.5$), CS^{FV}_{dsp} would predict different values of $\ln \gamma^{dsp}_{API}$ for them, just because of the difference in the M_{poly} and the corresponding x_{API} values, although the API-accessible surface area of the polymer (as an indicative measure of the probability of close surface API–polymer contacts leading to the dispersion interactions) is expected to be more or less the same in both equimass systems. To inspect this behavior, we plotted the average difference between CS^{FV} and CS^{FV}_{dsp} as a function of M_{poly} in Figure S12. A quite significant correlation of $R^2 = 0.86$ was found, indicating that the magnitude of the importance of the Margules-like $\ln \gamma^{dsp}_{API}$ decreases with M_{poly}. This may suggest that a re-formulation of the dispersion term would be needed, which would reflect the asymmetry of the polymeric systems by employing, *e.g.*, surface area fractions instead of mole fractions of the components. For clarity, this problem is only related to the composition dependence of $\ln \gamma^{dsp}_{i}$ and is not responsible for the negligible importance of the dispersion term in case of ASD.

For completeness, despite the negligible numerical difference between CS^{FV}_{dsp} and CS^{FV}, the solubilities obtained from CS^{FV} are higher than those from CS^{FV}_{dsp} in the majority of cases

(specifically, for 31 of the 35 systems and 86 % of the individual w_{API} data points calculated at T_{exp}). It is interesting that the remaining systems that do not follow this trend are again those with PVA as the polymer. However, even in these cases, the difference between w_{API} values predicted by $\mathrm{CS}^{\mathrm{FV}}$ and $\mathrm{CS}^{\mathrm{FV}}_{\mathrm{dsp}}$ is not larger than 1 ppm.

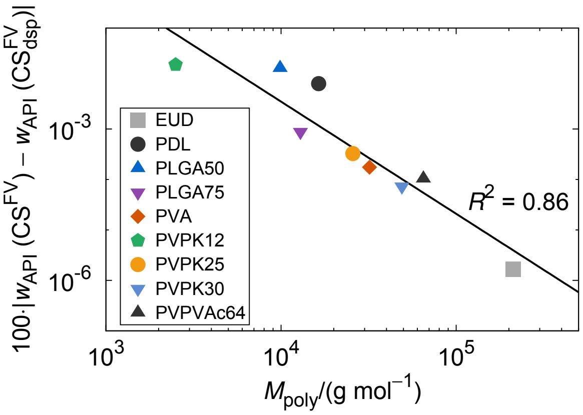

Figure S12: Average difference between $\mathrm{CS}^{\mathrm{FV}}$ and $\mathrm{CS}^{\mathrm{FV}}_{\mathrm{dsp}}$ as a function of polymer molar mass.

S10 Sensitivity Analysis: σ-Profiles of Polymers

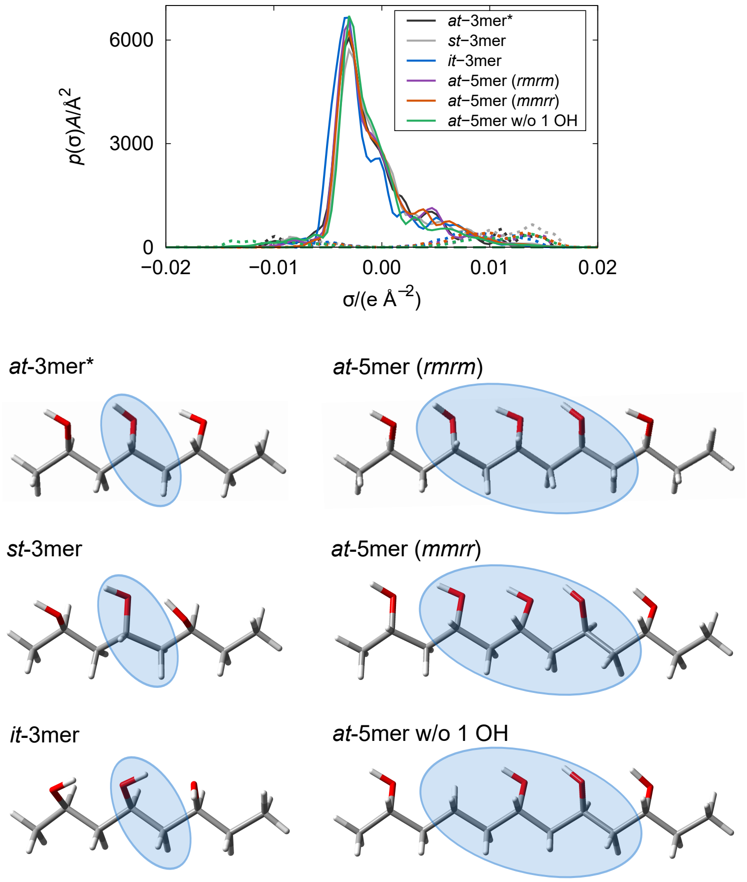

Figure S13: σ-profiles of virtual PVA molecules of 32 000 g mol^{-1} (726 units) derived from the different oligomer molecules shown in the lower panels. The shaded fragments represent the units whose properties were replicated (in the case of the pentamers, the central triad was replicated only 241 times). The asterisk (*) denotes the oligomer considered in the reference approach.

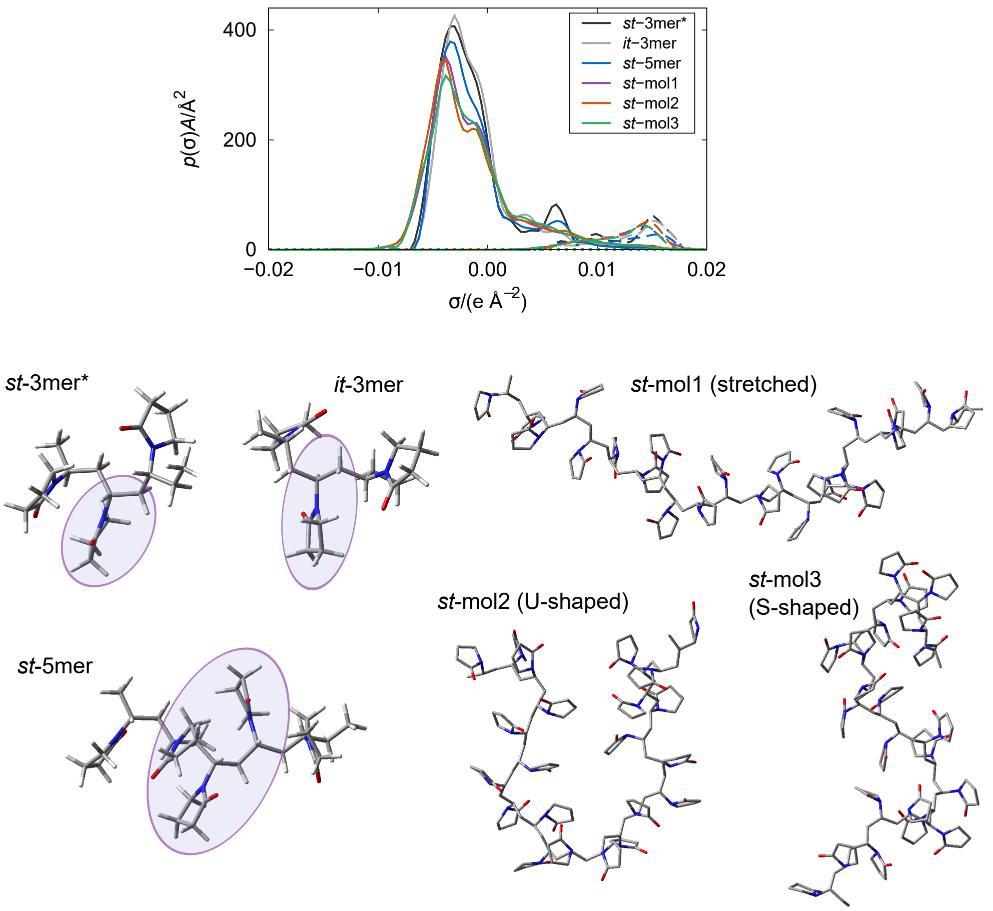

Figure S14: σ-profiles of (i) virtual PVPK12 molecules of $2500\,\mathrm{g\,mol^{-1}}$ (22 units) derived from the different oligomer molecules shown below (the shaded fragments represent the units whose properties were replicated; in the case of the pentamer, the central triad was replicated only 7 times) and (ii) three "real" liquid-phase PVPK12 molecules selected from an equilibrated MD simulation volume[18] at a temperature of 500 K (with the formula $C_{134}H_{204}N_{22}O_{22}$; 1338 electrons). The σ-profiles of these molecules were calculated directly from the molecules themselves, bypassing the replication method. Although these molecules only represent a sample of the numerous possible conformations that PVPK12 molecules can adopt in the liquid phase, they provide an indicative sample. Note that PVPK12 molecules, being relatively short compared to the other considered polymers, are not expected to adopt complicated coil-like shapes. Nevertheless, the considered molecules exhibit distinct conformational states. The asterisk (*) denotes the oligomer considered in the reference approach.

References

(1) Prudic, A.; Ji, Y. H.; Sadowski, G. Thermodynamic Phase Behavior of API/Polymer Solid Dispersions. *Mol. Pharmaceutics* **2014**, *11*, 2294–2304.

(2) Iemtsev, A.; Hassouna, F.; Klajmon, M.; Mathers, A.; Fulem, M. Compatibility of Selected Active Pharmaceutical Ingredients with Poly(D, L-Lactide-Co-Glycolide): Computational and Experimental Study. *Eur. J. Pharm. Biopharm.* **2022**, *179*, 232–245.

(3) Prausnitz, J. M.; Lichtenthaler, R. N.; de Azevedo, E. G. *Molecular Thermodynamics of Fluid-Phase Equilibria*, 3rd ed.; Prentice Hall: Upper Saddle River, NJ, 1999.

(4) Burghardt, W. R. Phase-Diagrams for Binary Polymer Systems Exhibiting Both Crystallization and Limited Liquid Liquid Miscibility. *Macromolecules* **1989**, *22*, 2482–2486.

(5) Byun, H.-S.; Lee, B.-S. Liquid-Liquid Equilibrium of Hydrogen Bonding Polymer Solutions. *Polymer* **2017**, *121*, 1–8.

(6) Amsterdam Modeling Suite (AMS) 2022.101, Software for Chemistry and Materials (SCM), Theoretical Chemistry, Vrije Universiteit. `http://www.scm.com`, accessed 2022-07-21.

(7) Klajmon, M. Investigating Various Parametrization Strategies for Pharmaceuticals within the PC-SAFT Equation of State. *J. Chem. Eng. Data* **2020**, *65*, 5753–5767.

(8) ChemSpider Database. `http://www.chemspider.com/Chemical-Structure.4330.html`, accessed 2022-09-28.

(9) Mathers, A.; Hassouna, F.; Malinová, L.; Merna, J.; Růžička, K.; Fulem, M. Impact of Hot-Melt Extrusion Processing Conditions on Physicochemical Properties of Amorphous Solid Dispersions Containing Thermally Labile Acrylic Copolymer. *J. Pharm. Sci.* **2020**, *109*, 1008–1019.

(10) Luebbert, C.; Huxoll, F.; Sadowski, G. Amorphous-Amorphous Phase Separation in API/Polymer Formulations. *Molecules* **2017**, *22*, 296.

(11) Zoller, P.; Walsh, D. *Standard Pressure-Volume-Temperature Data for Polymers*; Technomic Publishing Company, Inc.: Lancaster, PA, 1995.

(12) Knopp, M. M.; Olesen, N. E.; Holm, P.; Lobmann, K.; Holm, R.; Langguth, P.; Rades, T. Evaluation of Drug-Polymer Solubility Curves Through Formal Statistical Analysis: Comparison of Preparation Techniques. *J. Pharm. Sci.* **2015**, *104*, 44–51.

(13) Knopp, M. M.; Olesen, N. E.; Holm, P.; Langguth, P.; Holm, R.; Rades, T. Influence of Polymer Molecular Weight on Drug-Polymer Solubility: A Comparison between Experimentally Determined Solubility in PVP and Prediction Derived from Solubility in Monomer. *J. Pharm. Sci.* **2015**, *104*, 2905–2912.

(14) Iemtsev, A.; Zemánková, A.; Hassouna, F.; Mathers, A.; Klajmon, M.; Slámová, M.; Malinová, L.; Fulem, M. Ball Milling and Hot-Melt Extrusion of Indomethacin-L-Arginine-Vinylpyrrolidone-Vinyl Acetate Copolymer: Solid-State Properties and Dissolution Performance. *Int. J. Pharm.* **2022**, *613*, 121424.

(15) Bondi, A. A. *Physical Properties of Molecular Crystals, Liquids, and Glasses*; Wiley: New York, NY, 1968.

(16) Zhao, Y. H.; Abraham, M. H.; Zissimos, A. M. Fast Calculation of van der Waals Volume as a Sum of Atomic and Bond Contributions and Its Application to Drug Compounds. *J. Org. Chem.* **2003**, *68*, 7368–7373.

(17) Klajmon, M. Purely Predicting the Pharmaceutical Solubility: What to Expect from PC-SAFT and COSMO-RS? *Mol. Pharmaceutics* **2022**, *19*, 4212–4232.

(18) Klajmon, M.; Aulich, V.; Ludík, J.; Červinka, C. Glass Transition and Structure of Organic Polymers from All-Atom Molecular Simulations. *Ind. Eng. Chem. Res.* **2023**, *62*, 21437–21448.

(19) Pavliš, J.; Mathers, A.; Fulem, M.; Klajmon, M. Can Pure Predictions of Activity Coefficients from PC-SAFT Assist Drug–Polymer Compatibility Screening? *Mol. Pharmaceutics* **2023**, *20*, 3960–3974.

(20) Ge, K.; Huang, Y.; Ji, Y. Machine Learning with API/Polymer Interaction Mechanism: Prediction for Complex Phase Behaviors of Pharmaceuticals and Formulations. *Chin. J. Chem. Eng.* **2024**, *66*, 263–272.

(21) Mathers, A. *Prediction of Drug Solubility in Polymer: Combined Experimental and Computational Study*; Doctoral dissertation, University of Chemistry and Technology, Prague, 2022.

(22) Mathers, A.; Pechar, M.; Hassouna, F.; Fulem, M. API Solubility in Semi-Crystalline Polymer: Kinetic and Thermodynamic Phase Behavior of PVA-Based Solid Dispersions. *Int. J. Pharm.* **2022**, *623*, 121855.

(23) Mathers, A.; Hassouna, F.; Klajmon, M.; Fulem, M. Comparative Study of DSC-Based Protocols for API-Polymer Solubility Determination. *Mol. Pharmaceutics* **2021**, *18*, 1742–1757.

(24) Mathers, A. *Various API–Polymer Solubility Datasets Determined via Differential Scanning Calorimetry*; Unpublished raw data, University of Chemsitry and Technology, Prague, 2022.

(25) Gross, J.; Sadowski, G. Perturbed-Chain SAFT: An Equation of State Based on a Perturbation Theory for Chain Molecules. *Ind. Eng. Chem. Res.* **2001**, *40*, 1244–1260.

(26) Iemtsev, A.; Klajmon, M.; Hassouna, F.; Fulem, M. Effect of Copolymer Properties on the Phase Behavior of Ibuprofen-PLA/PLGA Mixtures. *Pharmaceutics* **2023**, *15*, 645.

(27) Mathers, A.; Pechar, M.; Hassouna, F.; Fulem, M. The Step-Wise Dissolution Method: An Efficient DSC-Based Protocol for Verification of Predicted API–Polymer Compatibility. *Int. J. Pharm.* **2023**, *648*, 123604.

(28) Hsieh, C.-M.; Lin, S.-T.; Vrabec, J. Considering the Dispersive Interactions in the COSMO-SAC Model for More Accurate Predictions of Fluid Phase Behavior. *Fluid Phase Equilib.* **2014**, *367*, 109–116.
